# Deciphering the genetic basis of resistance to soybean cyst nematode combining IBD and association mapping

**DOI:** 10.1007/s00122-023-04268-3

**Published:** 2023-03-13

**Authors:** Yu Tian, Delin Li, Xueqing Wang, Hao Zhang, Jiajun Wang, Lijie Yu, Changhong Guo, Xiaoyan Luan, Xinlei Liu, Hongjie Li, Jochen C. Reif, Ying-hui Li, Li-juan Qiu

**Affiliations:** 1 The National Key Facility for Gene Resources and Genetic Improvement/Key Laboratory of Crop Germplasm Utilization, Institute of Crop Sciences, Chinese Academy of Agricultural Science, Beijing, 100081 People’s Republic of China; 2 Soybean Research Institute, Heilongjiang Academy of Agricultural Sciences, Harbin, 150086 China; 3 Key Laboratory of Molecular Cytogenetics and Genetic Breeding, College of Life Science and Technology, Harbin Normal University, Heilongjiang Province, Harbin, China; 4 Department of Breeding Research, Leibniz Institute of Plant Genetics and Crop Plant Research (IPK), Gatersleben, Germany

## Abstract

**Key message:**

IBD analysis clarified the dynamics of chromosomal recombination during the ZP pedigree breeding process and identified ten genomic regions resistant to SCN race3 combining association mapping.

**Abstract:**

Soybean cyst nematode (SCN, *Heterodera glycines* Ichinohe) is one of the most devastating pathogens for soybean production worldwide. The cultivar Zhongpin03-5373 (ZP), derived from SCN-resistant progenitor parents, Peking, PI 437654 and Huipizhi Heidou, is an elite line with high resistance to SCN race3. In the current study, a pedigree variation map was generated for ZP and its ten progenitors using 3,025,264 high-quality SNPs identified from an average of 16.2 × re-sequencing for each genome. Through identity by decent (IBD) tracking, we showed the dynamic change of genome and detected important IBD fragments, which revealed the comprehensively artificial selection of important traits during ZP breeding process. A total of 2,353 IBD fragments related to SCN resistance including SCN-resistant genes *rhg1*, *rhg4* and *NSF*_RAN07_ were identified based on the resistant-related genetic paths. Moreover, 23 genomic regions underlying resistance to SCN race3 were identified by genome-wide association study (GWAS) in 481 re-sequenced cultivated soybeans. Ten common loci were found by both IBD tracking and GWAS analysis. Haplotype analysis of 16 potential candidate genes suggested a causative SNP (C/T, − 1065) located in the promoter of *Glyma.08G096500* and encoding a predicted TIFY5b-related protein on chr8 was highly correlated with SCN race3 resistance. Our results more thoroughly elucidated the dynamics of genomic fragments during ZP pedigree breeding and the genetic basis of SCN resistance, which will provide useful information for gene cloning and the development of resistant soybean cultivars using a marker-assisted selection approach.

**Supplementary Information:**

The online version contains supplementary material available at 10.1007/s00122-023-04268-3.

## Introduction

As one of the most economically relevant pathogens, soybean cyst nematode (SCN, *Heterodera glycines* Ichinohe) causes significant production problem in soybean [*Glycine max* (L.) Merr.] worldwide annually (Kim et al. 2016; Koenning and Wrather 2010). Of the all of nine races recorded in China, race 3 was the predominant one (Chen et al. 2001; Lu et al. 2006). Cultivation of resistant cultivars and appropriate crop rotation system with non-host crops has been shown to be the most effective and sustainable method of controlling damage caused by SCN in soybean production (Concibido et al. 2004; Meinhardt et al. 2021). To data, more than 500 resistant accessions have been identified from about 30,000 cultivated soybean accessions in the USDA Soybean Germplasm Collection and the Chinese National Soybean GeneBank (CNSGB) (Anand et al. 1988; Chen et al. 2006; Li et al. 2011; Ma et al. 2006), but few of them have been extensively incorporated into the improvement of modern cultivars with SCN resistance (Concibido et al. 2004; Li et al. 2011). As a result, most of the released varieties exploit single source of SCN resistance, about 95% of modern soybean varieties with resistance to SCN carry PI 88788 resistance (Zhou et al. 2021). Limited single-source resistance has led to an increasingly problem in the soybean SCN breeding with a narrow genetic basis resulting in a rapid loss of resistance and promoting the spread of the disease (Chowdhury et al. 2021; Niblack et al. 2008). To protect soybean production, it is necessary to develop resistant varieties with more horizontal or broad-spectrum resistance by pyramiding both major resistance genes and minor genes.

Resistance to SCN in soybean is a complex quantitative trait controlled by multiple loci/genes (Arelli et al. 1997; Concibido et al. 2004; Kim et al. 2016; Wu et al. 2009; Yue et al. 2001). Although more than 200 QTL (http://www.soybase.org/) may contribute to SCN resistance, only two major (*rhg1* and *Rhg4*) and two minor (*GmSNAP11* and *NSF*_RAN07_) loci had been cloned (Bayless et al. 2018; Cook et al. 2012; Lakhssassi et al. 2017; Liu et al. 2017, 2012; Tian et al. 2019). The remaining QTL always encompassed a large genomic region due to the limited recombination rate, which limited gene clone and marker-assisted selection. Recently, pedigree-based analysis (identity-by-descent mapping, IBD) has been shown to be an effective method for fine mapping quantitative traits (Huang et al. 2018; Kover et al. 2009; Ma et al. 2018). The resolution of IBD analysis depends on demographic history of the population and the strength of selection against trait-causing variants. Thus, this method can potentially help to overcome some of the limitations of current association and linkage mapping, such as difficulty in identifying rare variants in association mapping, or poor resolution in linkage mapping due to limited recombination rates in bi-parental populations (Bink et al. 2014; Zheng et al. 2017; Zhou et al. 2016). More importantly, the pedigree population has been subjected to continuous selection by breeders and is therefore an important source to “bridge” between QTLs and breeder (Yamamoto et al. 2010). A clear breeding history with a detailed pedigree makes it an ideal model for deciphering genetic architecture of modern breeding, which would uncover selected genome regions and the characterization of artificial selection (Zhou et al. 2016).

However, few studies have addressed the elucidating of genome-wide genetic architecture and identified the causal genes controlling complex quantitative traits during the pedigree breeding process in soybean. In this study, we re-sequenced and analyzed the ZP pedigree population to elucidate the dynamics of chromosomal recombination during the ZP pedigree breeding process and key IBD fragments or genes, and to identify and fine map genomic regions associated with resistance to SCN inherited from the three resistant progenitor parents Peking, PI 437654 and HPZHD. We then verified IBD fragments and detected additional genome regions underlying resistance to SCN race3 via the genome-wide association study (GWAS) of the diverse panel of 481 soybean germplasm accessions. By combining the IBD and GWAS analysis, we identified potential candidate genes involved in SCN race3 resistance to develop new cultivars with durable resistance to SCN using a marker-assisted selection approach.

## Material and methods

### Plant materials

**Fig. 1:**
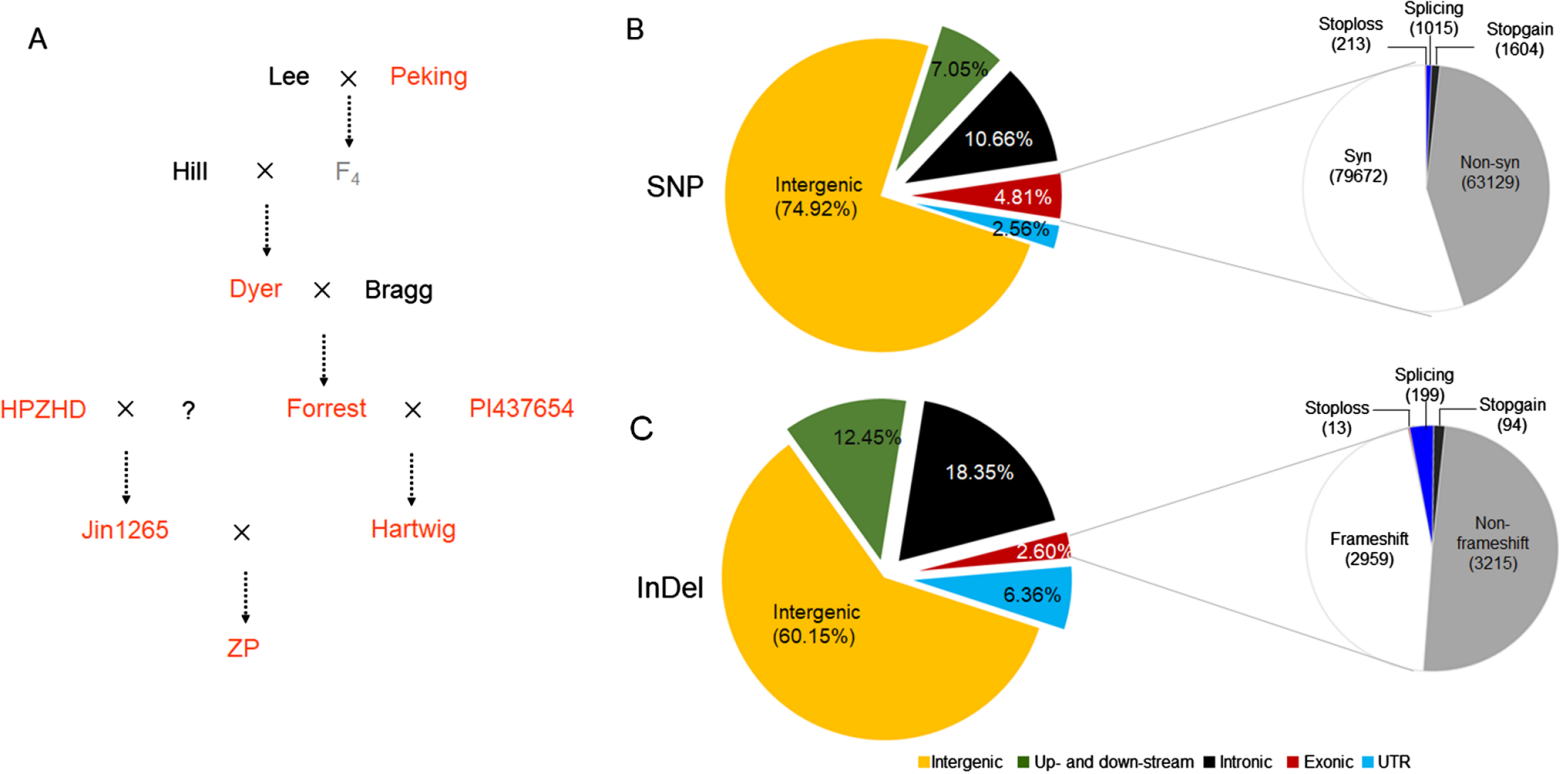
Identification and annotation of genetic variations in one family-based panel included 11 genealogical soybean genotypes. **A** The pedigree diagram of the ZP and its ten donor parents. The red characters indicate those genotypes resistance to SCN race3. The question mark represents the unknown parent. **B** Overall and large-effect SNPs. **C** Overall and large-effect InDels

Two types of panels with different genetic diversity were used in the present study (Supplementary Table S1). The family-based panel included 11 genealogical genotypes (Hill, Lee, Peking, Dyer, Bragg, Forrest, PI 437654, Hartwig, HPZHD, Jin1265, and ZP) (Fig. 1, Supplementary Table S1). The diversity panel consisted of 478 soybean accessions including 306 landraces and 172 improved cultivars and the three pedigree founders Peking, PI 437654, and HPZHD (Supplementary Table S1). This panel was obtained from 1,993 re-sequenced cultivated soybean (Supplementary Fig. S1), which were mainly selected from Chinese primary and applied core collections that represented the most genetic diversity of the Chinese Soybean Germplasm Collection (Li et al. 2022). For the family-based panel, five pedigree genotypes including Bragg, Dyer, Lee, Jin1265, and Hill were re-sequenced. Sequencing data of the remaining six genotypes were obtained from published studies (Supplementary Table S1) (Li et al. 2022; Tian et al. 2022).

### SCN bioassay

Family-based and association mapping population were screened for resistance to SCN race3 at Heilongjiang Academy of Agricultural Sciences in 2017 and 2018 using the published method (Li et al. 2016). Briefly, plants were placed in the greenhouse and field in soil infested with SCN race3. Resistant accessions Peking, Pickett, PI 90763, PI 88788 and the susceptible accession Lee and Hefeng 25 were planted as differential controls. For the greenhouse experiment, fifteen plants were grown in 3 autoclaved soil-filled PVC tubes (3 × 20 cm) plots with 5 plants per tubes representing each line or accession. Five-day soybean seedings were inoculated with about 1,000 hatched second-state juveniles (J2) of SCN HG Type 0 (race3) each and grown in a greenhouse under 16 h light/8 h dark at 25℃. Thirty days after post-inoculation, plants for each line or accession were randomly selected in a greenhouse to count the number of cysts on the root. Resistance was derived from three repeated screening for confirmation. For the field experiment, the SCN-infested field soil contained about 6,000 eggs (40 cysts) per 100 g of dry soil, and these materials were planted in a completely randomized design with 3 replications. Each row was 0.65 m wide and 1.5 m long with 0.05 m plant spacing. Thirty days after emergence, five plants in the center of each row were randomly selected to count the number of cysts on the roots of the plant and its rhizosphere soil of approximately 150 g collected using two nested 710- and 250-um aperture sieves. Female index (FI) (Schmitt and Shannon 1992) was calculated by dividing the number of cysts on an experimental line by the number of cysts on the SCN susceptible accession Hefeng 25 and multiplying by 100%, which was used to evaluate the response of each accession to the SCN race3.

### Sample collection and library preparation

Genomic DNA of the five pedigree genotypes Bragg, Dyer, Lee, Jin1265, and Hill was extracted from leaves using the cetyltrimethylammonium bromide (CTAB) method (Doyle and Doyle 1990) with a total amount of 1.5 μg DNA per sample used as input material for the DNA sample preparations. Sequencing libraries were prepared using Truseq Nano DNA HT Sample preparation Kit (Illumina, USA) following manufacturer’s recommendations. Briefly, the DNA sample was fragmented by sonication to a size of 350 bp, and then the DNA fragments were polished at the ends, A-tailed, and ligated with the full-length adapter for Illumina sequencing with further PCR amplification. Further, the PCR products were purified (AMPure XP system) and libraries were analyzed for size distribution by Agilent2100 Bioanalyzer and quantified using real-time PCR.

### Genome sequencing and mapping

Whole genomes of the five genotypes were re-sequenced based on the Illumina Hiseq2500 platform, and we obtained a total of 194.15 Gb raw data for the 11 genealogical soybeans, combining with the seven previously re-sequenced genomes (Supplementary Table S2). A total of 192.36 Gb (~ 16.24-fold per genotype) high-quality paired-end reads for the 11 genealogical soybeans was generated. The high-quality paired-end reads were mapped to the reference soybean genome Williams 82.a2.v1 using the BWA (Li and Durbin 2009) software with the command ‘mem − t 10 − k 32’. Then, we used SAMtools (Li et al. 2009) package to convert SAM alignment to BAM format. Finally, for each genotype, an average 97.9% of reads across the 11 genealogical soybeans were mapped to the soybean reference genome Williams 82.a2.v1 (Supplementary Table S2).

### Genetic variation identification

After mapping, genetic variations for the 11 genealogical soybeans were detected using the package SAMtools with the ‘mpileup’ program, of which the parameters ‘ − m 2 − F 0.002 − d 1000’. The variants were filtered for downstream analysis by requiring a minimum coverage of 4 and a maximum coverage of 200, a minimum RMS mapping quality of 20 and no gaps present within a 3 bp window. Consequently, an average of 2,120,346 (from 1,714,366 to 2,839,873 for each line) high credible SNPs (missing rate < 20%) was kept for further analysis (Supplementary Table S3). Meanwhile, we also identified an average of 192,032 (from 145,708 to 263,554) insertion and deletions (InDels, 1–15 bp) (Supplementary Table S4). All SNPs and InDels were annotated using the package ANNOVAR (Wang et al. 2010).

### IBD detection

The IBD fragments between ZP and the remaining 10 genealogical soybeans were detected by Beagle version4 program (window = 500, overlap = 250, ibdlod = 3, ibdtrim = 40; Browning and Browning 2013). Furthermore, the ancestral origin of IBD fragments of ZP derived from different progenitor were calculated based on the pedigree relationship.

### Population structure analysis and linkage disequilibrium

A principal component analysis (PCA) of 481 accessions in the diversity panel was performed using GCTA software (Yang et al. 2011). The population structure was analyzed using admixture software (Alexander et al. 2009) with parameters *k* = 2 to 10. Linkage disequilibrium (LD) was calculated using PLINK1.9 (Version1.90) software (Purcell et al. 2007). The LD decay of whole genome was calculated and drawn based on the average *r*^2^ value for the distance from 0 to 1000 kb using PopLDdecay (Zhang et al. 2019).

### Genome-wide association study

Association analysis was performed using Tassel software package v5 (Bradbury et al. 2007) with a mixed linear model (MLM) model (K + Q). Kinship matrices were calculated using centered_IBS method in Tassel software to determine the relatedness among association population. The significance threshold was defined by the false discovery rate (FDR) method (*P* < 0.01). Based on the mean LD decay distance of whole genome, genes located in 100 kb genomic region of both sides of each peak SNP were considered as the candidate genes.

## Results

### The genetic variations of 11 genealogical genotypes

To elucidate the genetic architecture of ZP, 11 genealogical genotypes were re-sequenced and combined with previously reported re-sequencing profiles (Fig. 1A, Supplementary Table S1) (Li et al. 2022; Tian et al. 2022). A total of 192.36 Gb high-quality sequence data were aligned against the soybean reference genome Williams 82.a2.v1 after adapter removal and quality trimming. On average, 97.9% of cleaned reads were mapped to the reference genome. Overall, the effective depth across these sequenced soybean genomes ranged from 14.3 × (PI 437654) to 19.9 × (Bragg) coverage, with an average of 16.2 × for each genome (Supplementary Table S2). Compared with reference genome Williams 82.a2.v1, a total of 3,025,264 high-quality single nucleotide polymorphisms (SNPs) were detected in the 11 genomes (Fig. 1B, S2, Supplementary Table S3), ranging from 1.71 million for Forrest to 2.84 million for HPZHD (Supplementary Table S3). A proportion of 74.92% (2,266,503) of all SNPs were found in the intergenic regions, 10.66% (322,434) in introns, 2.56% (77,388) in UTRs and 4.81% (144,618) in gene coding regions (Fig. 1B). Besides, 63,129 non-synonymous SNPs and 2,832 SNPs that resulted in a gain (1,604)/loss (213) of stop codon, a change of splice donor or acceptor sites (splicing sites 1,015) in protein-coding regions were defined as large-effect genetic variations (Fig. 1B, Supplementary Table S3). Moreover, 238,414 InDels were cataloged, including 6,480 large-effect InDels leading to mutations of splicing sites (199), frame-shift (2,959), non-frame-shift (3,215), as well as 94 gain and 13 loss of stop codon (Fig. 1C, Supplementary Table S4).

Pairwise IBS values among the 11 accessions ranged from 0.49 (Bragg and PI 437654) to 0.91 (Forrest and Hartwig) (Supplementary Table S5). IBS estimates between offspring and their direct parents were generally larger than those of indirect parents. For example, the IBS between ZP and its direct parents Jin1265 and Hartwig were 0.83 and 0.77, respectively, which was higher than that between the indirect Forrest (0.71), PI 437654 (0.62) and HPZHD (0.59). This suggests that the genetic relationship of pedigree was correct and could be used in the following analysis.

### Genomic constitution of ZP

**Fig. 2:**
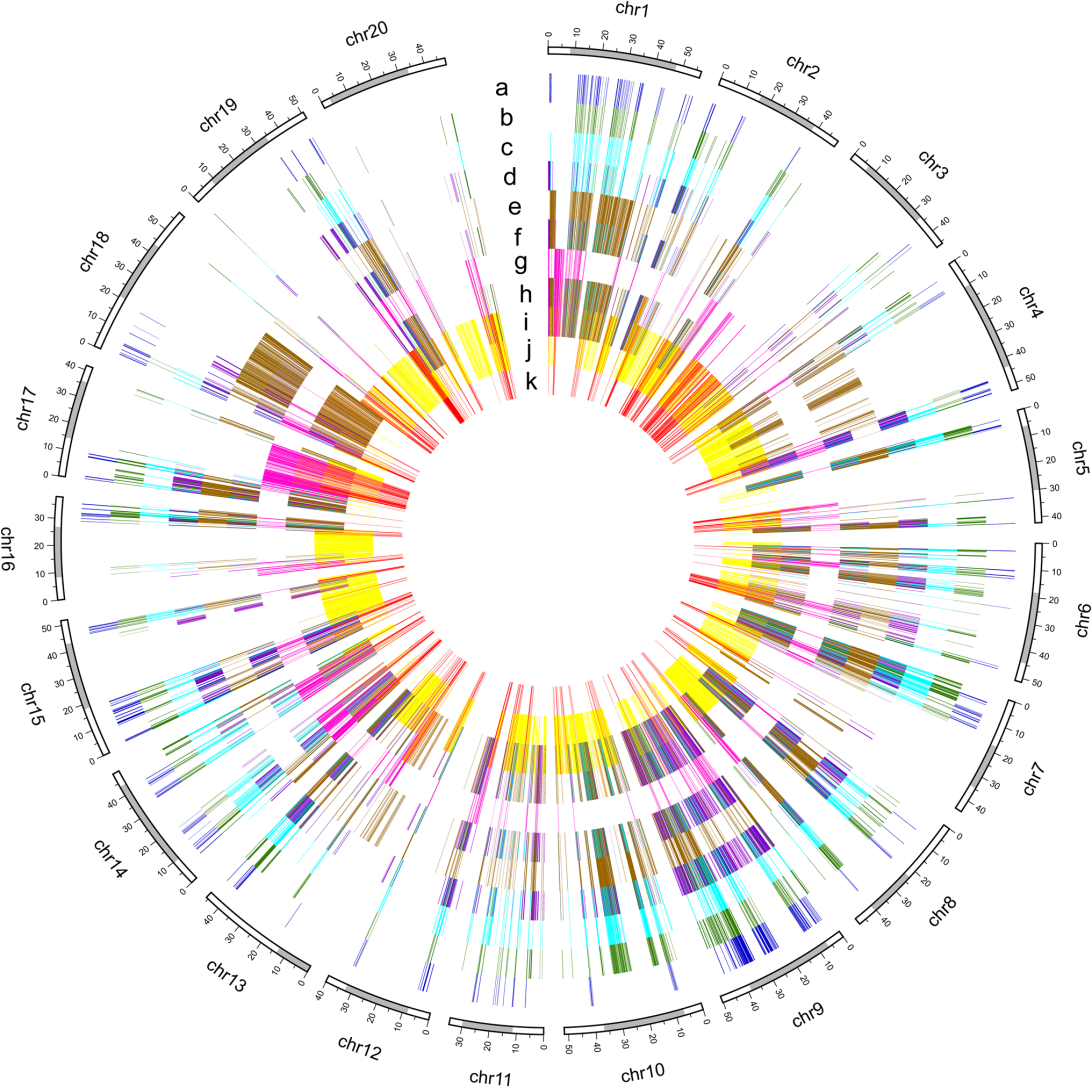
Gene flow of ZP. The IBD fragments from different donor parents are shown in different colors. **a** Peking, **b** Lee, **c** Hill, **d** Dyer, **e** Bragg, **f** Forrest, **g** PI 437654, **h** Hartwig, **i** ZP, **j** Jin1265, **k** HPZHD. The horizontal axis (top) indicates 20 soybean chromosomes, and the grey region represents the pericentromeric region

IBD analysis suggested that 41.17% of the genomic sequence of ZP was from the male parent Jin1265 and 32.87% from the female parent Hartwig (Fig. 2, Supplementary Fig. S3A). The haplotypes of the remaining genetic regions (25.96%) were untraceable or were caused by historic recombination (Supplementary Fig. S3A, Supplementary Table S6). Furthermore, we traced the ancestral origin of these fragments in the pedigree and found that of genomic sequence of 41.17% from Jin1265, 7.62% came from HPZHD. Of the 32.87% of the genomic sequence of ZP contributed by Hartwig, 8.38% was inherited from PI 437654, 3.45% from Peking and 27.76% from three susceptible parents including Bragg (14.55%), Hill (6.74%) and Lee (6.47%), respectively (Supplementary Fig. S3B). As expected, 19 cloned genes controlling important traits were also identified from IBD fragments selected in at least two consecutive breeding generation (Supplementary Table S7), such as determinate habit (*Dt1*), seed oil content (*GmSWEET39*), seed weight (*GmPP2C* and *GmST05*), nodule number (*GmNNL1*), SCN resistance (*NSF*_RAN07_, *GmSHMT08* and *GmSNAP18*). The result indicated that these traceable IBD fragments played an important role during ZP breeding process.

### Identification of the IBD fragments related to the SCN resistance

In the ZP pedigree (Fig. 1)A, seven parents, including Peking, PI 437654, HPZHD, Dyer, Forrest, Hartwig and Jin1265, showed high resistance to SCN race3, with female index (FI) ranged from 0 to 7.75% (Supplementary Table S1). The three parents Hill, Lee and Bragg were highly susceptible to SCN race3 and their FI ranged from 86.4% to 100% (Supplementary Table S1). Based on the pedigree, SCN resistance of ZP was derived from Peking, PI 437654 and HPZHD.

**Fig. 3:**
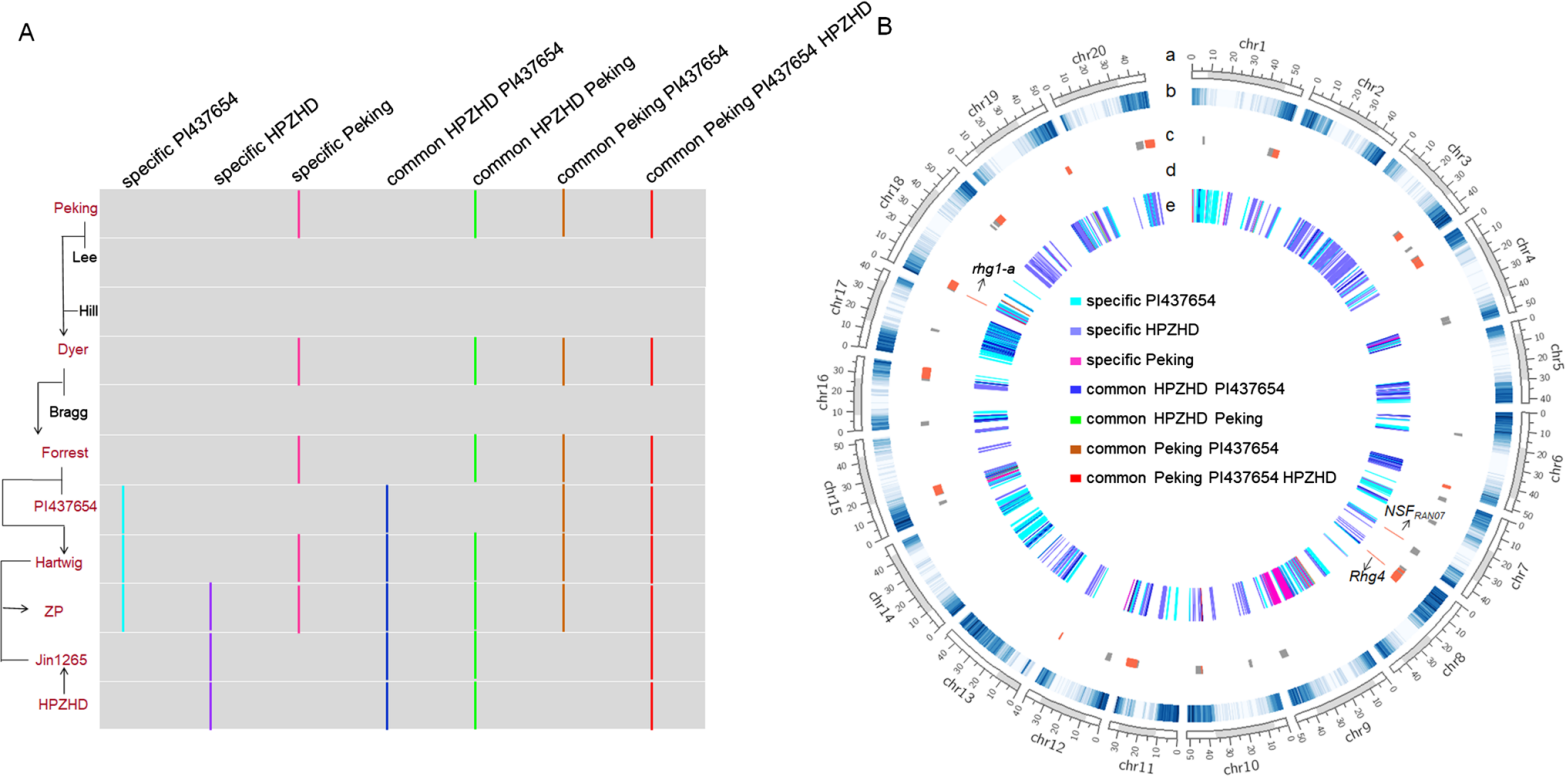
Identification of IBD fragments correlated to SCN resistance in the ZP pedigree. **A** Derivation pathway of the identification of IBD fragments related to resistance. The briefly transitive graph on the left indicates pedigree relationship, and the red character represents resistant lines. Vertical lines on the right represent the inheritable IBD fragments by seven genetic pathways from three ancestral resistance parents. **B** Verification and distribution of IBD fragments related to resistance. a. Chromosome. b. Gene density. c. Reported SCN resistance QTL overlapping with candidate resistant related-IBD fragments. Overlapped region among different QTL is indicated by red color. d. Cloned SCN resistance genes overlapping with candidate resistant related-IBD fragments. e. Distribution of IBD fragments on genome. Different colors indicate derivation pathways of IBD fragments. The grey region on chromosome represents pericentromeric regions

The IBD fragments inherited from the three resistant progenitors Peking, PI 437654, and HPZHD were analyzed to identify the IBD fragments controlling resistance to SCN through different genetic pathways in the pedigree (Fig. 3A). As a result, 2,353 IBD fragments were detected covering a genome region of 114.27 Mb. Eighteen of them (0.76%) came from the consensus fragments of the three resistant progenitors, 543 (23.08%) from the consensus fragments of any two resistant progenitors and 1,792 (76.16%) from the specific fragments of any resistant progenitors (Fig. 3B, Supplementary Table S8). These traceable IBD fragments related to SCN resistance by ZP pedigree analysis were compared with reported QTL for resistance to different SCN races to deeply explore correlation between IBD fragments and SCN resistance. These IBD fragments were overlapped with 150 reported QTL for resistance to different races (QTL region < 5 Mb), distributed across 16 chromosomes, except for chr2, chr5, chr13 and chr14 (Fig. 3B, Supplementary Table S9). Three resistant genes *GmSNAP18*, *GmSHMT08* and *NSF*_RAN07,_ were also detected within the inherited IBD fragments underlying SCN race3 resistance (Fig. 3B). We found that the average sizes of traceable IBD fragments were much smaller than reported QTL (Supplementary Fig. S4A). For instance, at the *rhg1* loci, a 102.3 kb common IBD fragment on chr18 derived from Peking, PI 437654 and HPZHD contained only 17 genes (Supplementary Fig. S4B), whereas the previous QTL region spanned average 1.26 Mb genomic region. These narrowed genomic regions identified by pedigree analysis will be helpful in mapping genes involved in SCN resistance.

### Genome-wide association study for resistance to SCN race3

The FI of 418 accessions in the diversity panel varied continuously (Supplementary Fig. S5). A total of 147 accessions exhibited high resistance (FI < 10) and 46 accessions showed moderate resistance (10 < FI < 30). To verify and fine map the genome regions underlying resistance to SCN race3, a diversity panel containing 4,096,160 SNPs (MAF ≥ 0.05, missing rate < 0.2 and heterozygosity < 0.1) were used for GWAS analysis. The overall LD decay distance for this panel was about 100 kb when *r*^*2*^ dropped to half of its maximum value (Supplementary Fig. S6). Principal component analysis and population structure classified these accessions into three subclades associating with the geographic distribution patterns, North region (NR), Huanghuai region (HR) and South region (SR), respectively (Supplementary Fig. S7, S8).

**Fig. 4:**
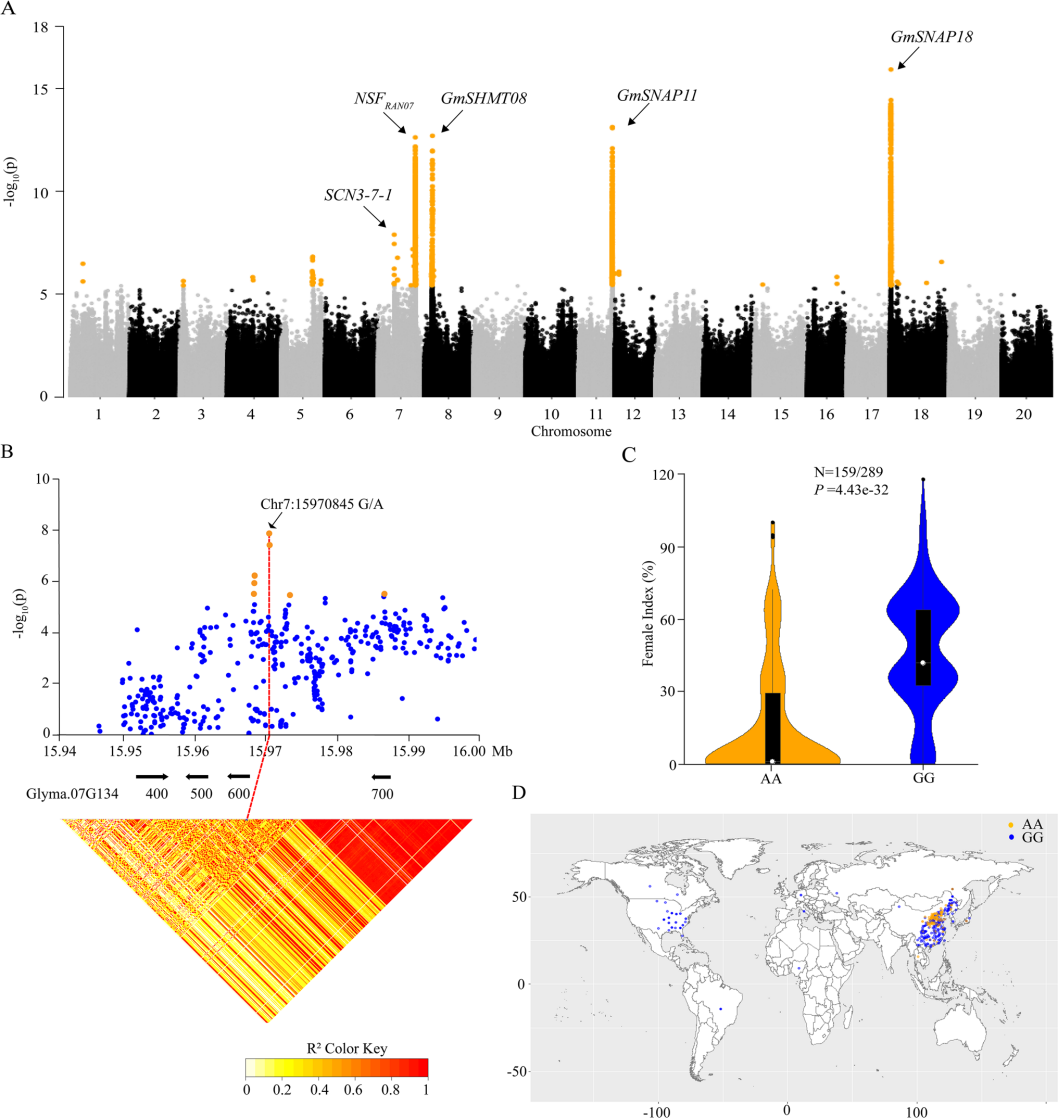
Identification of genetic loci controlling resistance to SCN race3 based on genome-wide association analysis. **A** Whole genome-wide Manhattan plot. Yellow dots indicate the SNPs significantly associated with SCN races resistance (FDR, *P* < 0.01). **B** Regional Manhattan plot (top) and LD heatmap (bottom) for locus *SCN3-7–1*. The corresponding annotated genes located in the regional plot are drawn in black arrows in the middle part. The bottom part shows the LD block of region plot. **C** The difference of FI between two allele chr7:15,970,845 genotypes (GG/AA) in soybean accessions. *P* value was determined by one-way ANOVA analysis. **D** Geographical distribution of SNP chr7:15,970,845 genotypes

Using a standard mixed linear model, GWAS analysis identified 23 genome regions significantly associated with SCN race3 resistance (FDR, *P* < 0.01), which were located on 11 chromosomes (chr.) (Fig. 4A, S9, Supplementary Table S10). Among them, four regions on chr7, chr8, chr11, and chr18 covered the four cloned resistant genes *NSF*_RAN07_, *GmSHMT08*, *GmSNAP11* and *GmSNAP18* and explained 13.01%, 12.83%, 13.55% and 16.98% of the phenotypic variances, respectively (Supplementary Table S10). In addition, some minor loci on chr1, chr3, chr4, chr5, chr7, chr12, chr15, chr16 and chr18 were also determined (Fig. 4A). Among them, a novel genomic region *SCN3-7–1* on chr7 was detected and explained 7.95% of the phenotypic variance (Fig. 4A; Supplementary Table S10). The most strongly associated SNP (chr7:15,970,845, G/A) was located in a 22.1 kb LD block region involving in four annotate genes (*Glyma.07G134400*, *Glyma.07G134500*, *Glyma.07G134600* and *Glyma.07G134700*) (Fig. 4B). The average FI of the 159 entries carrying the AA allele (15.6%) was significantly (*P* = 4.43e-32) lower than that of the 289 entries carrying GG allele (46.5%) (Fig. 4C). The resistant AA allele was mainly prevalent in the Huanghuai region of China, where a large number of resistant accessions were identified (Fig. 4D).

We analyzed the genetic background of ZP and its three resistant progenitors Peking, PI 437654, and HPZHD based on 23 significant loci identified by association analysis and found that ZP carried only 60.7% resistant alleles via the pedigree breeding, although three resistant progenitors contained 95.7% resistant alleles (Supplementary Table S11). This indicated that about 35% of the resistance loci had been underutilized during ZP breeding process. For example, Peking, PI 437654 and HPZHD carried the resistant allele of *SCN3-7–1*; however, ZP lost the resistant allele of S*CN3-7–1*.

### Identification of the candidate genes against SCN race3 by integrating IBD and GWAS analysis

Ten overlapping loci covering a genomic interval of 1.21 Mb genome interval were identified by comparing the genomic region of IBD and GWAS analysis (Supplementary Table S12). A total of 144 annotated genes were identified in these ten genomic regions, including three cloned genes, *NSF*_RAN07_, *GmSHMT08*, and *GmSNAP18* (Supplementary Table S13). Further, 425 SNPs in the gene coding regions of 106 genes were detected using 481 re-sequenced accessions. Among those, 220 SNPs located in 79 genes were large-effect SNP changing the encoding amino acid sequence, containing 215 non-synonymous SNP, 4 stop gain and 1 stop loss (Supplementary Table S13). Further, gene expression patterns change of these putatively resistant genes in response to the SCN race3 inoculation were further analyzed using previously published data (Klink et al. 2009; Li et al. 2018; Miraeiz et al. 2020; Wan et al. 2015). A total of 16 genes showed significantly differential expression profiles and half of them also carried large-effect variations (Supplementary Fig. S10–12, Supplementary Table S13). These genes were predicted to be involved in various biological functions (https://www.soybase.org/) (Supplementary Table S13), which could be vital to deeply explore the genetic basis of resistance to SCN race3.

**Fig. 5:**
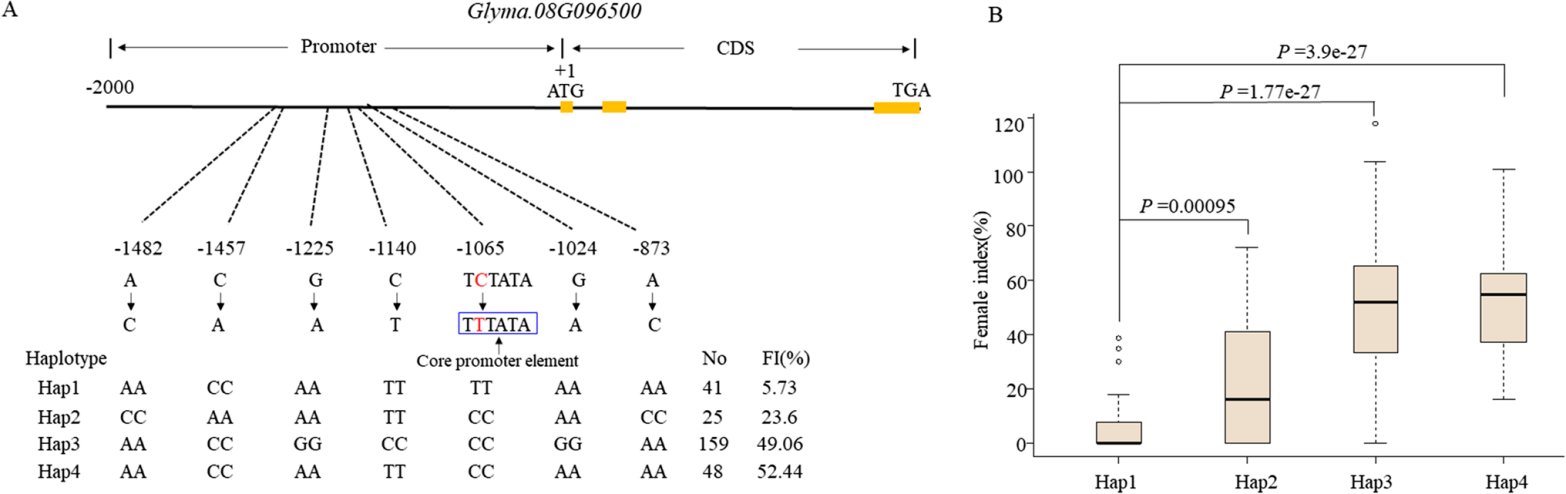
Genetic variations of gene *Glyma.08G096500* underlying resistance to SCN race3. **A** Allelic variations and haplotypes of *Glyma.08G096500* including promoter (upstream 2-kb of start codon) and coding sequence (CDS) from diversity panel. Hap1-Hap4 indicate four different haplotypes of *Glyma.08G096500*. The yellow boxes represent exons. The sequence in blue boxes represented a core promoter element (TATA-box) predicted by Plant CARE. **B** Comparison of FI among soybean accessions with different haplotype. The central bold line within the box indicates the median; box edges represent the upper and lower quantiles; whiskers shows the 1.5 interquartile range and points represent outliers. *P*-values were determined by one-way ANOVA analysis

The closest paralog *AT1G30135* of *Glyma.08G096500* encoded a jasmonate-zim-domain protein 8 associated with resistance to *Botrytis cinerea* and *Alternaria brassicicola* by jasmonate-mediated plant defense in *Arabidopsis thaliana* (Chen et al. 2021). The jasmonic acid-involved signaling was also involved in reaction to SCN race3 infection (Zhang et al. 2017)*. Glyma.08G096500* showed highly expression in root (https://phytozome-next.jgi.doe.gov/) and encoded a predicted TIFY5b-related protein that played vital roles in plant abiotic and biotic stress responses (Liu et al. 2022). These results strengthen the deduction that *Glyma.08G096500* was putatively functional gene resistant to SCN race3. Considering the expression level of *Glyma.08G096500* was significantly changed after SCN race3 inoculation (Wang et al., 2015), we examined the allelic variation of the *Glyma.08G096500* (promoter and coding region) in the diversity panel and identified a total of seven SNP which formed four haplotypes (Fig. 5A). Using the Plant CARE software (http://bioinformatics.psb.ugent.be/webtools/plantcare/html/), SNP (C/T, − 1065) was predicted to change the core promoter element (TATA-box) that play a critical role in the regulation of transcription (Deng and Roberts 2005) and response to stress (Basehoar et al. 2004), which distinguished H1 from the remaining three haplotypes (Fig. 5A). Haplotype analysis indicated that the average FI of the 41 entries carrying Hap1 (5.73%) was significantly lower than that of the entries carrying Hap2 (25, 23.6%), Hap3 (159, 49.06%) and Hap4 (48, 52.44%), respectively (Fig. 5B).

## Discussion

### IBD fragments play the essential role in ZP breeding process

Pedigree analysis is an effective method to reveal the dynamic change of the genome during crop breeding. In recent years, several publications have used re-sequenced elite pedigree populations with well-defined genetic paths to analyze the inference of core genomic fragments and their association with key traits during the breeding process (Chen et al. 2017; Han et al. 2020; Ma et al. 2018; Wu et al. 2016; Yamamoto et al. 2010; Zheng et al. 2017). However, few studies have re-sequenced soybean pedigrees to elucidate genome-wide genetic characters during the soybean breeding process. In the current study, we re-sequenced the ZP pedigree panel, in which resistance to SCN is one of the major improved traits, and elucidated the dynamic change of the genome during the breeding process. The proportion of genome constitution contributed to ZP by all progenitor varied from 3.45% (Peking) to 14.55% (Bragg). It was worth noticing that Bragg, a susceptible line to SCN race3, was the major contributor that played a vital role in the ZP breeding process, rather than the resistant ancestral parents. This indicates that intensive selection for other some traits such as grain yield, plant height and maturity time occurred in addition to SCN resistance.

A total of 19 genes controlling important traits were identified in these traceable IBD fragments selected in at least two consecutive breeding generation (Supplementary Table S7). Interestingly, the crucial genes of soybean flowering system, *E1*, *E3*, *Tof12* and *GmGBP1* were located in these IBD fragments selected in at least two continued breeding generation. Therefore, selection on these genes to expand the adaptation range adaptative of soybean cultivars appears to be an important component to soybean breeding. As expected, many reported genes controlling other important traits were also identified, including *GmLHY1a* for plant height and internode length (Cheng et al. 2019), *GmSWEET39* for seed oil content (Miao et al. 2019), and *GmST05* and *GmPP2C* for seed weight (Duan et al. 2022; Lu et al. 2017) (Supplementary Table S7). The traceable IBD fragments subjected to artificial selection provide an opportunity to explore the genetic change of genome during pedigree breeding. However, the function of the abundant IBD fragments still remains obscure. Reported QTL underlying important traits allow us to further hypothesize the possible function of IBD fragments. By comparing the position of IBD region and published QTL, a large number of QTL controlling important traits were overlapped with these traceable IBD segments (Supplementary Table S14), indicating that these IBD fragments may be closely associated with these traits. However, further studies are needed to validate these hypotheses.

### Verification of IBD fragments controlling resistance to SCN race3 by QTL mapping and GWAS

IBD tracing identified IBD fragments based on block identity between ZP and its parents in the pedigree with clear relationships. By comparing resistant and susceptible genotypes in the ZP pedigree, the resistant related-IBD fragments were defined. However, the generations of ZP pedigree and related parents were limited in current study, resulting in certain proportion of IBD fragments resistant to SCN possibly being false positives. Therefore, it seems reasonable to confirm these identified IBD fragments. QTL mapping and GWAS are two effective approaches to identify genetic loci underlying traits of interest from parental segregation group and diverse germplasms. We had conducted QTL mapping for resistance to SCN race3 using a RIL population derived from the ZP and cultivar Zhonghuang13 (susceptible to SCN race3) previously, and identified three QTL loci *SCN3-1*, *SCN3-11* and *SCN3-18* (Li et al. 2016; Yang et al. 2020), which also had been identified in the IBD analysis. However, the other two known resistant genes *Rhg4* and *NSF*_RAN07_ identified in the IBD analysis were not mapped in the RIL population, possibly due to the limited coverage of the genetic linkage map and the major-effect QTL, which limited the identification of other QTL such as *E1* (Lu et al. 2016) and the genetic interaction among different QTL in the RIL population. To overcome the limitation of a single method such as QTL mapping, GWAS analysis was performed to verify the loci underlying resistance to SCN race3. Of them, ten loci detected by GWAS for SCN race3 resistance were overlapped with inherited IBD fragment from the defined genetic paths associated with resistance to SCN (Supplementary Table S12). Furthermore, 354 resistant-related IBD fragments were overlapped by the reported QTL underlying resistance to different SCN race (Supplementary Table S9). This indicates that inheritable IBD fragments are genetic sources with resistance to different SCN race. For example, a novel *qSCN10* conferring broad-spectrum resistance was identified and mapping to 379 kb genomic region on chr10 from a soybean accession PI 567516C (Zhou et al. 2021). The region overlaps with the IBD fragment chr10:42,798,271–42,866,566 obtained from specific HPZHD (Supplementary Table S9). A resistant-related NBS–LRR protein encoding gene *Glyma.10G196700* located in the overlapping region of IBD and fine mapping can be identified as candidate gene for *qSCN10*. These results suggested that the joint IBD, QTL mapping and GWAS approaches can help in cross-validation and exploring deeply genetic basis of complex traits.

### Identification of novel candidate loci/genes underlying resistance to SCN race3

Two major resistance genes, *rhg1* and *Rhg4*, have been used widely in development of a large number of resistant cultivars in recent decades (Bayless et al. 2018; Patil et al. 2019; Zhou et al. 2021). However, over-reliance on limited numbers of resistant genes has led to the emergence of virulent SCN populations that are able to reproduce on these resistant cultivars (Gardner et al. 2017; Meinhardt et al. 2021). There is now an urgent need to identify novel sources of SCN resistance for overcome the genetic diversification of the SCN race. In the current study, a total of ten loci were discovered by combining IBD and GWAS analyses. We compared the ten common genomic regions obtained by IBD analysis and GWAS with reported QTL and QTN collected from Soybase (https://soybase.org/) and published studies (Supplementary Table S15, S16). Of them, five loci were located in or overlapped with the reported regions; the remaining five loci were not overlapped with these reported regions and defined as novel loci. Promising candidate genes were analyzed using differential gene expressions between resistant and susceptible accessions derived from studies and homologous gene comparison. For example, a TIFY 5b-related protein encoding gene located on chr8, *Glyma.08G096500* (homologous to *AT1G30135* in *Arabidopsis*) was found to be responsible for resistance to SCN race3 in both IBD and association analysis. *AT1G30135* was associated with jasmonate-mediated plant defense in *Arabidopsis thaliana* (Chen et al. 2021). Haplotype analysis showed that a SNP (C/T, − 1065) in *Glyma.08G096500* was significantly correlated with resistance to SCN race3 and was predicted to result in the change of core promoter element (TATA-box) (Fig. 5A). This core promoter element may be important for regulating the expression level of *Glyma.08G096500* based on its different expression level after inoculation of SCN race3 from Wan et al. 2015*.*

Additionally, a novel locus *SCN3-7–1* on chr7 (SNP chr7:15,970,845) was significantly associated with resistance to SCN race3. However, when tracing this locus in the ZP pedigree, we found that all three ancestral parents Peking, PI 437654 and HPZHD carried the resistant AA allele, but it was not passed on to the next generation (Supplementary Fig. S13). Interestingly, the proportion of the AA allele in association panel decreased from landraces (49.8%) to improved cultivars (10.9%) (Supplementary Fig. S14). Thus, although the soybean accessions carrying the AA allele were resistant to SCN race3, this allele was not selected by breeders. We speculated that this phenomenon may be explained by linkage drags with resulting in poor performance for other important traits such as grain yield. Therefore, we further analyzed the reported QTL surrounding *SCN3-7–1* (https://www.soybase.org/) and identified QTL controlling resistance to *Spodoptera litura*, resistance to *Sclerotinia sclerotiorum*, seed calcium content, and seed weight (Supplementary Table S17). In the *SCN3-7–1* region, a total of four predicted genes (*Glyma.07G134400*, *Glyma.07G134500*, *Glyma.07G134600* and *Glyma.07G134700*) were identified based on the reference genome Williams 82.a2.v1. In accordance with previously reported transcriptional responses to SCN race3 at 0, 3 and 8 days post-inoculation (Wan et al. 2015), *Glyma.07G134500* encoding a predicted heat shock protein DnaJ was constitutively down-regulated (*P* < 0.05) in the two resistant lines PI 437654 and PI 567516C compared with the susceptible cultivar Magellan at 3 and 8 days post-inoculation (Supplementary Fig. S15), which was considered as candidate gene underlying the *SCN3-7–1* locus. In conclusion, the predicted candidate genes are promising candidates for further exploring the genetic basis of resistance to SCN.

## Supplementary Information

Below is the link to the electronic supplementary material.

Supplementary file1 (TIF 16515 KB)

Supplementary file2 (TIF 876 KB)

Supplementary file3 (TIF 1007 KB)

Supplementary file4 (TIF 2706 KB)

Supplementary file5 (TIF 203 KB)

Supplementary file6 (TIF 195 KB)

Supplementary file7 (TIF 281 KB)

Supplementary file8 (TIF 569 KB)

Supplementary file9 (TIF 1549 KB)

Supplementary file10 (TIF 383 KB)

Supplementary file11 (TIF 533 KB)

Supplementary file12 (TIF 76 KB)

Supplementary file13 (TIF 838 KB)

Supplementary file14 (TIF 111 KB)

Supplementary file15 (TIF 280 KB)

Supplementary file16 (XLSX 3669 KB)

## Data Availability

All whole-genome sequencing data for the family-based panel were deposited into Sequence Read Archive (SRA) under BioProject accession PRJNA835908. For the diversity panel, sequencing data were extracted from the Genome Sequence Archive at the BIG Data Center, Beijing Institute of Genomics, Chinese Academy of Sciences (http://bigd.big.ac.cn/), under the accession number PRJCA003894.

## References

[CR1] Alexander DH, Novembre J, Lange K (2009). Fast model-based estimation of ancestry in unrelated individuals. Genome Res.

[CR2] Anand SC, Gallo KM, Baker IA, Hartwig EE (1988). Soybean plant introductions with resistance to races 4 or 5 of soybean cyst nematode. Crop Sci.

[CR3] Arelli APR, Wilcox JA, Myers O, Gibson PT (1997). Soybean germplasm resistant to races 1 and 2 of *Heterodera glycines*. Crop Sci.

[CR4] Bayless AM, Zapotocny RW, Grunwald DJ, Amundson KK, Diers BW, Bent AF (2018). An atypical N-ethylmaleimide sensitive factor enables the viability of nematode-resistant *Rhg1* soybeans. Proc Natl Acad Sci USA.

[CR63] Basehoar AD, Zanton SJ, Pugh BF (2004). Identification and Distinct Regulation of Yeast TATA Box-Containing Genes. Cell.

[CR5] Bink MC, Jansen J, Madduri M, Voorrips RE, Durel CE, Kouassi AB, Laurens F, Mathis F, Gessler C, Gobbin D, Rezzonico F, Patocchi A, Kellerhals M, Boudichevskaia A, Dunemann F, Peil A, Nowicka A, Lata B, Stankiewicz-Kosyl M, Jeziorek K, Pitera E, Soska A, Tomala K, Evans KM, Fernández-Fernández F, Guerra W, Korbin M, Keller S, Lewandowski M, Plocharski W, Rutkowski K, Zurawicz E, Costa F, Sansavini S, Tartarini S, Komjanc M, Mott D, Antofie A, Lateur M, Rondia A, Gianfranceschi L, van de Weg WE (2014). Bayesian QTL analyses using pedigreed families of an outcrossing species, with application to fruit firmness in apple. Theor Appl Genet.

[CR6] Bradbury PJ, Zhang Z, Kroon DE, Casstevens TM, Ramdoss Y, Buckler ES (2007). TASSEL: software for association mapping of complex traits in diverse samples. Bioinformatics.

[CR7] Browning BL, Browning SR (2013). Improving the accuracy and efficiency of identity-by-descent detection in population data. Genetics.

[CR8] Chen PS, Qi JS, Wang SH, Hu QY (2001). Studies on identification and monitoring of physiologic variation of *Heterodera glycines* in China. Acta Phytopathologica Sinica.

[CR9] Chen YW, Wang DC, Arelli P, Ebrahimi M, Nelson RL (2006). Molecular marker diversity of SCN-resistant sources in soybean. Genome.

[CR10] Chen SX, Lin ZC, Zhou DG, Wang CR, Li H, Yu RB, Deng HC, Tang XY, Zhou SC, Deng XW, He H (2017). Genome-wide study of an elite rice pedigree reveals a complex history of genetic architecture for breeding improvement. Sci Rep.

[CR11] Chen LG, Zhang LP, Xiang SY, Chen YL, Zhang HY, Yu DQ (2021). The transcription factor WRKY75 positively regulates jasmonate-mediated plant defense to necrotrophic fungal pathogens. J Exp Bot.

[CR12] Cheng Q, Dong LD, Su T, Li TY, Gan ZR, Nan HY, Lu SJ, Fang C, Kong LP, Li HY, Hou ZH, Kou K, Tang Y, Lin XY, Zhao XH, Chen LY, Liu BH, Kong FJ (2019). CRISPR/Cas9-mediated targeted mutagenesis of *GmLHY* genes alters plant height and internode length in soybean. BMC Plant Biol.

[CR13] Chowdhury IA, Yan G, Plaisance A, Markell S (2021). Characterization of virulence phenotypes of soybean cyst nematode (*Heterodera glycines*) populations in North Dakota. Phytopathology.

[CR14] Concibido VC, Diers BW, Arelli PR (2004). A decade of QTL mapping for cyst nematode resistance in soybean. Crop Sci.

[CR15] Cook DE, Lee TG, Guo XL, Melito S, Wang K, Bayless AM, Wang J, Hughes TJ, Willis DK, Clemente TE, Diers BW, Jiang JM, Hudson ME, Bent AF (2012). Copy number variation of multiple genes at *Rhg1* mediates nematode resistance in soybean. Science.

[CR16] Deng W, Roberts SG (2005). A core promoter element downstream of the TATA box that is recognized by TFIIB. Genes Dev.

[CR17] Doyle JJ, Doyle JL (1990). Isolation of plant DNA from fresh tissue. Focus.

[CR18] Duan ZB, Zhang M, Zhang ZF, Liang S, Fan L, Yang X, Yuan YQ, Pan Y, Zhou GA, Liu SL, Tian ZX (2022). Natural allelic variation of *GmST05* controlling seed size and quality in soybean. Plant Biotech J.

[CR19] Gardner M, Heinz R, Wang JY, Mitchum MG (2017). Genetics and adaptation of soybean cyst nematode to broad spectrum soybean resistance. G3 Genes Genomes Genet.

[CR20] Han ZG, Hu Y, Tian Q, Cao YW, Si AJ, Si ZF, Zang YH, Xu CY, Shen WJ, Dai F, Liu X, Fang L, Chen H, Zhang TZ (2020). Genomic signatures and candidate genes of lint yield and fibre quality improvement in upland cotton in Xinjiang. Plant Biotech J.

[CR21] Huang J, Li J, Zhou J, Wang L, Yang SH, Hurst LD, Li WH, Tian DC (2018). Identifying a large number of high-yield genes in rice by pedigree analysis, whole-genome sequencing, and CRISPR-Cas9 gene knockout. Proc Natl Acad Sci USA.

[CR22] Kim KS, Vuong TD, Qiu D, Robbins RT, Shannon JG, Li ZL, Nguyen HT (2016). Advancements in breeding, genetics, and genomics for resistance to three nematode species in soybean. Theor Appl Genet.

[CR23] Klink VP, Hosseini P, Matsye P, Alkharouf NW, Matthews BF (2009). A gene expression analysis of syncytia laser microdissected from the roots of the glycine max (soybean) genotype PI 548402 (Peking) undergoing a resistant reaction after infection by *Heterodera glycines* (soybean cyst nematode). Plant Mol Biol.

[CR24] Koenning SR, Wrather JA (2010). Suppression of soybean yield potential in the continental united states by plant diseases from 2006 to 2009. Plant Health Prog.

[CR25] Kover PX, Valdar W, Trakalo J, Scarcelli N, Ehrenreich IM, Purugganan MD, Durrant C, Mott R (2009). A multiparent advanced generation inter-cross to fine-map quantitative traits in *Arabidopsis thaliana*. PLoS Genet.

[CR26] Lakhssassi N, Liu SM, Bekal S, Zhou Z, Colantonio V, Lambert K, Barakat A, Meksem K (2017). Characterization of the soluble NSF attachment protein gene family identifies two members involved in additive resistance to a plant pathogen. Sci Rep.

[CR27] Li H, Durbin R (2009). Fast and accurate short read alignment with burrows-wheeler transform. Bioinformatics.

[CR28] Li H, Handsaker B, Wysoker A, Fennell T, Ruan J, Homer N, Marth G, Abecasis G, Durbin R (2009). The sequence alignment/map format and SAMtools. Bioinformatics.

[CR29] Li YH, Qi XT, Chang RZ, Qiu LJ, Sudaric A (2011). Evaluation and utilization of soybean germplasm for resistance to cyst nematode in China. Soybean molecular aspects of breeding.

[CR30] Li YH, Shi XH, Li HH, Reif JC, Wang JJ, Liu ZX, He S, Yu BS, Qiu LJ (2016). Dissecting the genetic basis of resistance to soybean cyst nematode combining linkage and association mapping. Plant Genome.

[CR31] Li S, Chen Y, Zhu XF, Wang YY, Jung KH, Chen LJ, Xuan YH, Duan YX (2018). The transcriptomic changes of Huipizhi Heidou (*Glycine max*), a nematode-resistant black soybean during *Heterodera glycines* race 3 infection. J Plant Physiol.

[CR32] Li YH, Qin C, Wang L, Jiao C, Hong H, Tian Y, Li Y, Xing G, Wang J, Gu Y, Gao X, Li D, Li H, Liu Z, Jing X, Feng B, Zhao T, Guan R, Guo Y, Liu J, Yan Z, Zhang L, Ge T, Li X, Wang X, Qiu H, Zhang W, Luan X, Han Y, Han D, Chang R, Guo Y, Reif JC, Jackson SA, Liu B, Tian SL, Qiu LJ (2022). Genome-wide signatures of geographic expansion and breeding process in soybean. Sci China Life Sci.

[CR33] Liu SM, Kandoth PK, Warren SD, Yeckel G, Heinz R, Alden J, Yang C, Jamai A, El-Mellouki T, Juvale PS (2012). A soybean cyst nematode resistance gene points to a new mechanism of plant resistance to pathogens. Nature.

[CR34] Liu SM, Kandoth PK, Lakhssassi N, Kang J, Colantonio V, Heinz R, Yeckel G, Zhou Z, Bekal S, Dapprich J (2017). The soybean *GmSNAP18* gene underlies two types of resistance to soybean cyst nematode. Nat Commun.

[CR35] Liu YL, Zheng L, Jin LG, Liu YX, Kong YN, Wang YX, Yu TF, Chen J, Zhou YB, Chen M, Wang FZ, Ma YZ, Xu ZS, Lan JH (2022). Genome-wide analysis of the soybean TIFY family and identification of *GmTIFY10e* and *GmTIFY10g* response to salt stress. Front Plant Sci.

[CR36] Lu WG, Gai JY, Li WD (2006). Sampling survey and identification of races of soybean cyst nematode (*Heterodera glycines* Ichinohe) in Huang-Huai Valleys. Agric Sci China.

[CR37] Lu SJ, Li Y, Wang JL, Nan HY, Cao D, Li XM, Shi DN, Fang C, Shi XY, Yuan XH, Jun A, Liu BH, Kong FJ (2016). Identification of additional QTLs for flowering time by removing the effect of the maturity gene *E1* in soybean. J Integr Agr.

[CR38] Lu X, Xiong Q, Cheng T, Li QT, Liu XL, Bi YD, Li W, Zhang WK, Ma B, Lai YC, Du WG, Man WQ, Chen SY, Zhang JS (2017). A PP2C-1 allele underlying a quantitative trait locus enhances soybean 100-seed weight. Mol Plant.

[CR39] Ma YS, Wang WH, Wang LX, Ma FM, Wang PW, Chang RZ, Qiu LJ (2006). Genetic diversity of soybean and the establishment of a core collection focused on resistance to soybean cyst nematode. J Integr Plant Biol.

[CR40] Ma XF, Wang ZY, Li W, Zhang YZ, Zhou XJ, Liu YG, Ren ZY, Pei XY, Zhou KH, Zhang WS, He KL, Zhang F, Liu JF, Ma WY, Xiao GH, Yang DG (2018). Resequencing core accessions of a pedigree identifies derivation of genomic segments and key agronomic trait loci during cotton improvement. Plant Biotech J.

[CR41] Meinhardt C, Howland A, Ellersieck M, Scaboo A, Diers B, Mitchum M (2021). Resistance gene pyramiding and rotation to combat widespread soybean cyst nematode virulence. Plant Dis.

[CR42] Miao L, Yang SN, Zhang K, He JB, Wu C, Ren YH, Gai JY, Li Y (2019). Natural variation and selection in *GmSWEET39* affect soybean seed oil content. New Phytol.

[CR43] Miraeiz E, Chaiprom U, Afsharifar A, Karegar A, Drnevich JM, Hudson ME (2020). Early transcriptional responses to soybean cyst nematode HG Type 0 show genetic differences among resistant and susceptible soybeans. Theor Appl Genet.

[CR44] Niblack TL, Colgrove AL, Bond JP (2008). Shift in virulence of soybean cyst nematode is associated with use of resistance from PI 88788. Plant Health Prog.

[CR45] Patil GB, Lakhssassi N, Wan J, Song L, Zhou Z, Klepadlo M, Vuong TD, Stec AO, Kahil SS, Colantonio V (2019). Whole-genome re-sequencing reveals the impact of the interaction of copy number variants of the *rhg1* and *Rhg4* genes on broad-based resistance to soybean cyst nematode. Plant Biotech J.

[CR46] Purcell S, Neale B, Todd-Brown K, Thomas L, Ferreira MAR, Bender D, Maller J, Sklar P, Bakker PIWD, Daly MJ (2007). PLINK: a tool set for whole-genome association and population-based linkage analyses. Am J Hum Genet.

[CR47] Schmitt DP, Shannon G (1992). Differentiating soybean responses to *Heterodera glycines* races. Crop Sci.

[CR48] Tian Y, Liu B, Shi XH, Reif JC, Guan RX, Li YH, Qiu LJ (2019). Deep genotyping of the gene *GmSNAP* facilitates pyramiding resistance to cyst nematode in soybean. Crop J.

[CR49] Tian Y, Yang L, Lu HF, Zhang B, Li YF, Liu C, Ge TL, Liu YL, Han JN, Li YH, Qiu LJ (2022). QTL analysis for plant height and fine mapping of two environmentally stable QTLs with major effects in soybean. J Integr Agr.

[CR50] Wan JR, Vuong T, Jiao YQ, Joshi T, Zhang HX, Xu D, Nguyen HT (2015). Whole-genome gene expression profiling revealed genes and pathways potentially involved in regulating interactions of soybean with cyst nematode (*Heterodera glycines* Ichinohe). BMC Genom.

[CR51] Wang K, Li MY, Hakonarson H (2010). ANNOVAR: functional annotation of genetic variants from high-throughput sequencing data. Nucleic Acids Res.

[CR52] Wu XL, Blake S, Sleper DA, Shannon JG, Cregan P, Nguyen HT (2009). QTL, additive and epistatic effects for SCN resistance in PI 437654. Theor Appl Genet.

[CR53] Wu X, Li Y, Fu J, Li X, Li C, Zhang D, Shi Y, Song Y, Li Y, Wang TY (2016). Exploring identity-by-descent segments and putative functions using different foundation parents in maize. PLoS ONE.

[CR54] Yamamoto T, Nagasaki H, Yonemaru J-i, Ebana K, Nakajima M, Shibaya T, Yano M (2010). Fine definition of the pedigree haplotypes of closely related rice cultivars by means of genome-wide discovery of single-nucleotide polymorphisms. BMC Genom.

[CR55] Yang J, Lee SH, Goddard ME, Visscher PM (2011). GCTA: a tool for genome-wide complex trait analysis. Am J Hum Genet.

[CR56] Yang L, Tian Y, Liu YL, Reif JC, Li YH, Qiu LJ (2020). QTL mapping of *qSCN3-1* for resistance to soybean cyst nematode in soybean line Zhongpin 03–5373. Crop J.

[CR57] Yue P, Arelli PR, Sleper DA (2001). Molecular characterization of resistance to *Heterodera glycines* in soybean PI 438489B. Theor Appl Genet.

[CR58] Zhang HY, Kjemtrup-Lovelace S, Li CB, Luo Y, Chen LP, Song BH (2017). Comparative RNA-seq analysis uncovers a complex regulatory network for soybean cyst nematode resistance in wild soybean (*Glycine soja*). Sci Rep.

[CR59] Zhang C, Dong SS, Xu JY, He WM, Yang TL (2019). PopLDdecay: a fast and effective tool for linkage disequilibrium decay analysis based on variant call format files. Bioinformatics.

[CR60] Zheng XF, Li LZ, Liang F, Tan CJ, Tang SZ, Yu SB, Diao Y, Li SC, Hu ZL (2017). Pedigree-based genome re-sequencing reveals genetic variation patterns of elite backbone varieties during modern rice improvement. Sci Rep.

[CR61] Zhou DG, Chen W, Lin ZC, Chen HD, Wang CR, Li H, Yu RB, Zhang FY, Zhen G, Yi JL, Li KH, Liu YG, Terzaghi W, Tang XY, He H, Zhou SC, Deng XW (2016). Pedigree-based analysis of derivation of genome segments of an elite rice reveals key regions during its breeding. Plant Biotech J.

[CR62] Zhou LJ, Song L, Lian Y, Ye H, Usovsky M, Wan JR, Vuong TD, Nguyen HT (2021). Genetic characterization of *qSCN10* from an exotic soybean accession PI 567516C reveals a novel source conferring broad-spectrum resistance to soybean cyst nematode. Theor Appl Genet.

