# Supplementary figures and images for: Deciphering the genetic basis of resistance to soybean cyst nematode combining IBD and association mapping
Source: Theor Appl Genet. 2023 Mar 13;136(3):50. doi: 10.1007/s00122-023-04268-3 (PMC10011322; doi:10.1007/s00122-023-04268-3)

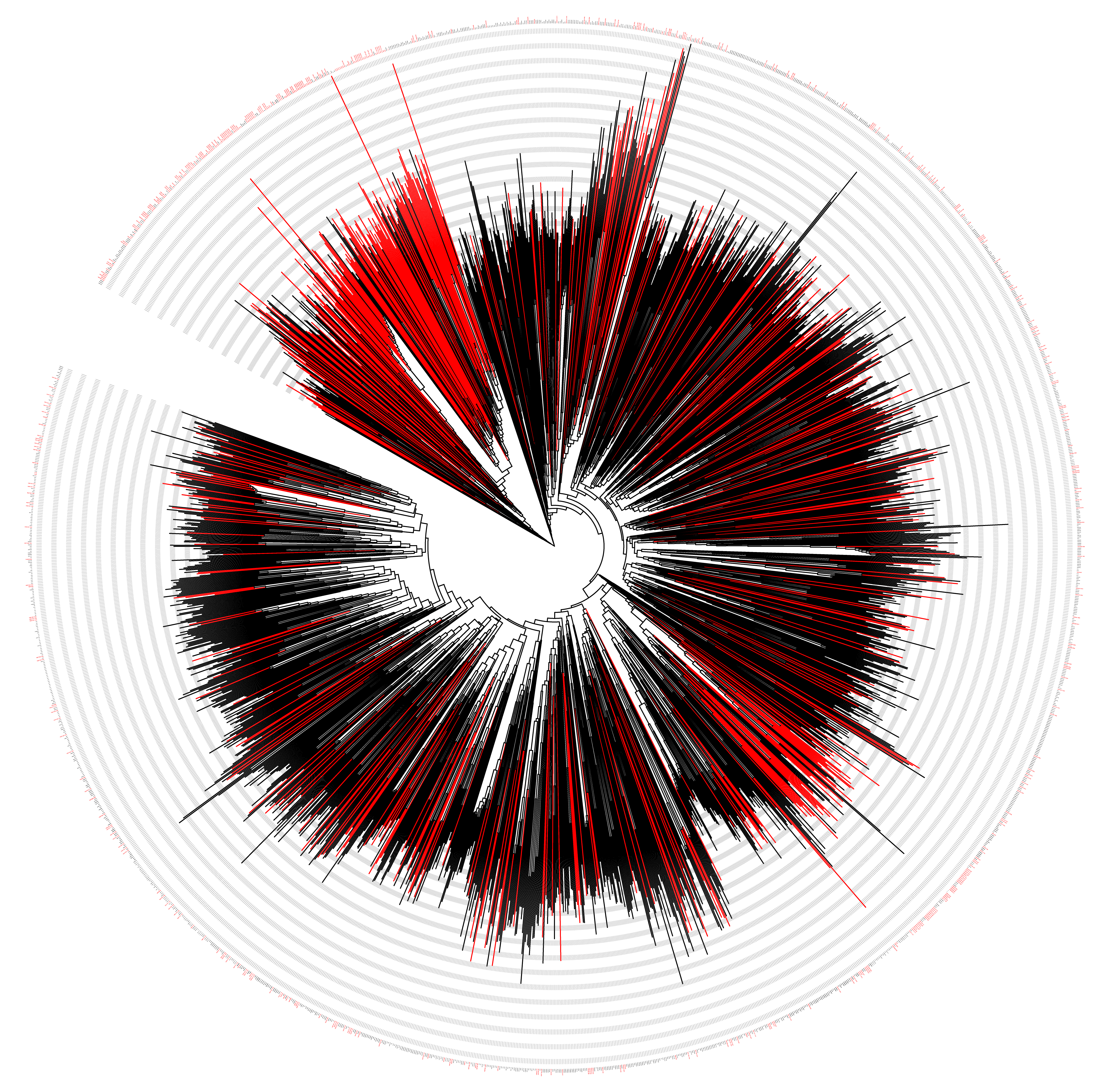

Supplement: Supplementary file 1 — Supplementary file1 (TIF 16515 KB) [file 122_2023_4268_MOESM1_ESM.tif]

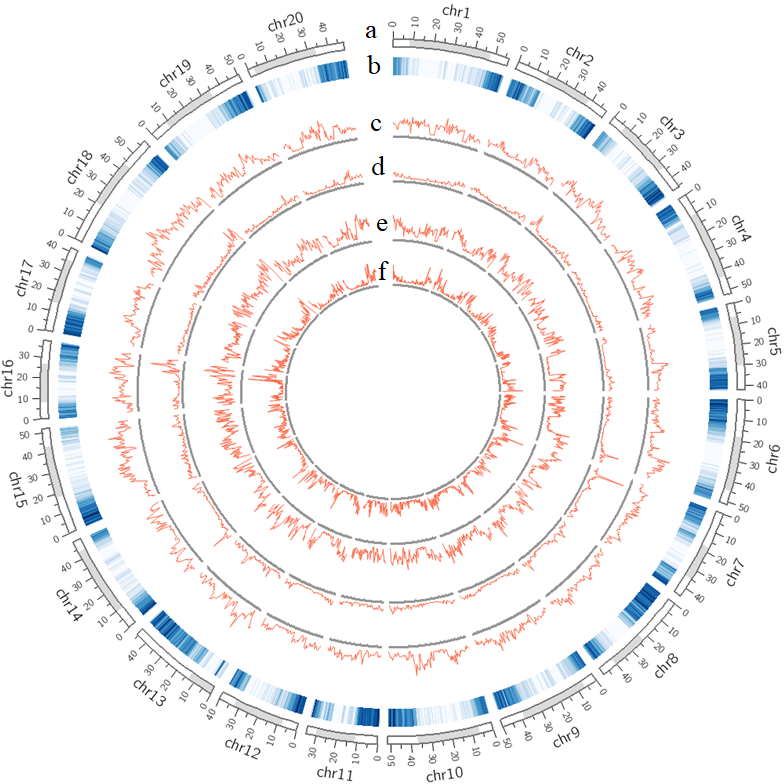

Supplement: Supplementary file 2 — Supplementary file2 (TIF 876 KB) [file 122_2023_4268_MOESM2_ESM.tif]

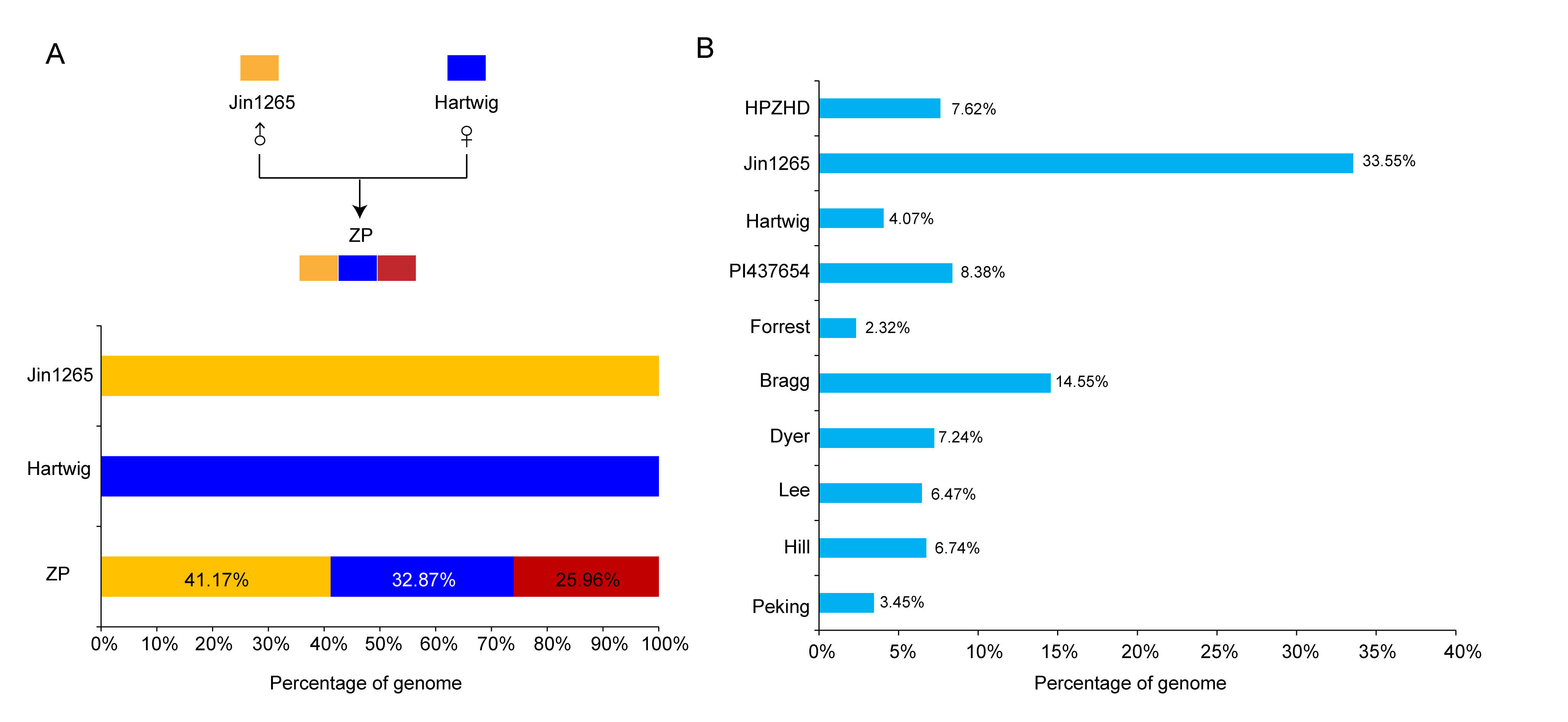

Supplement: Supplementary file 3 — Supplementary file3 (TIF 1007 KB) [file 122_2023_4268_MOESM3_ESM.tif]

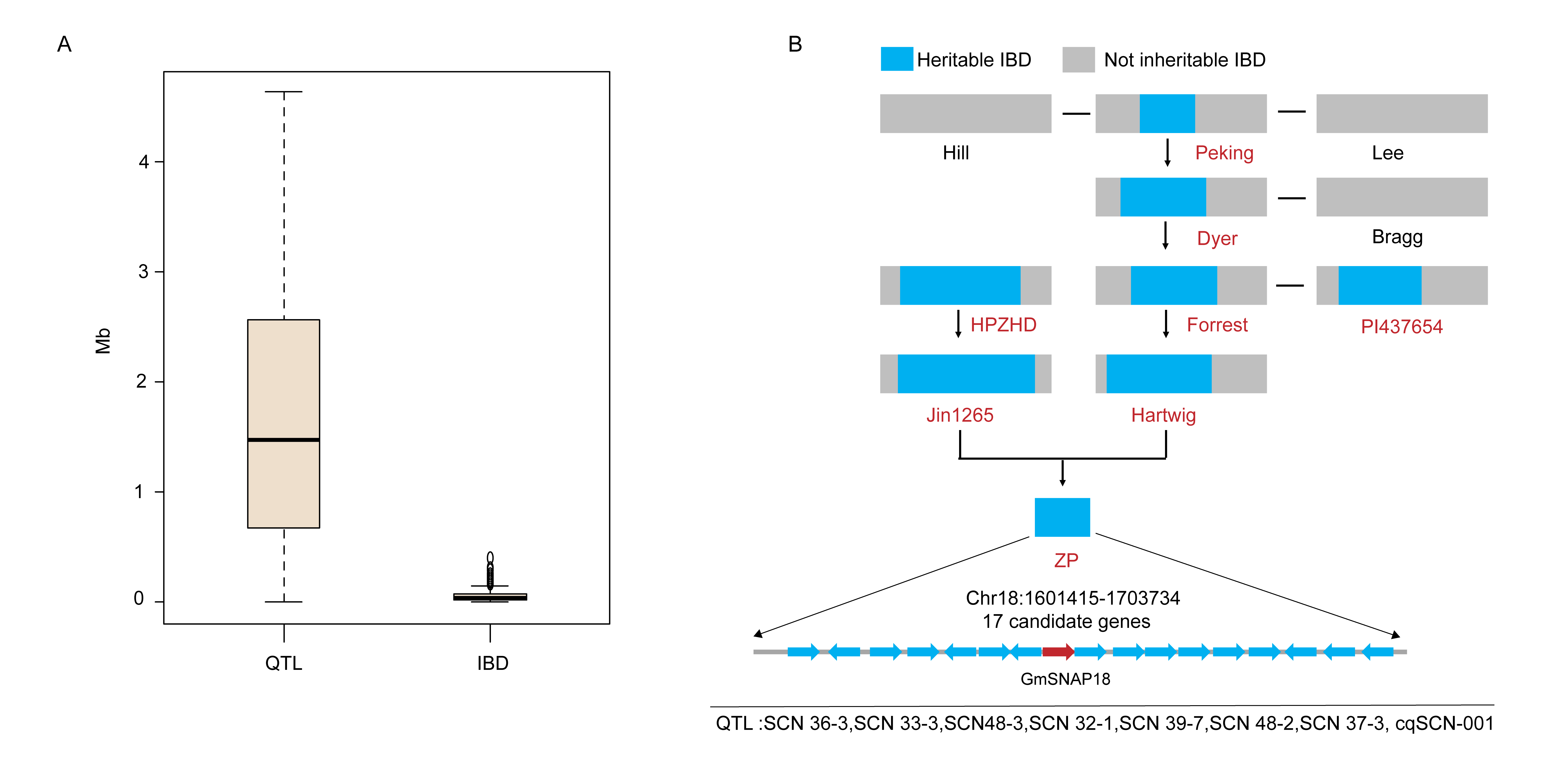

Supplement: Supplementary file 4 — Supplementary file4 (TIF 2706 KB) [file 122_2023_4268_MOESM4_ESM.tif]

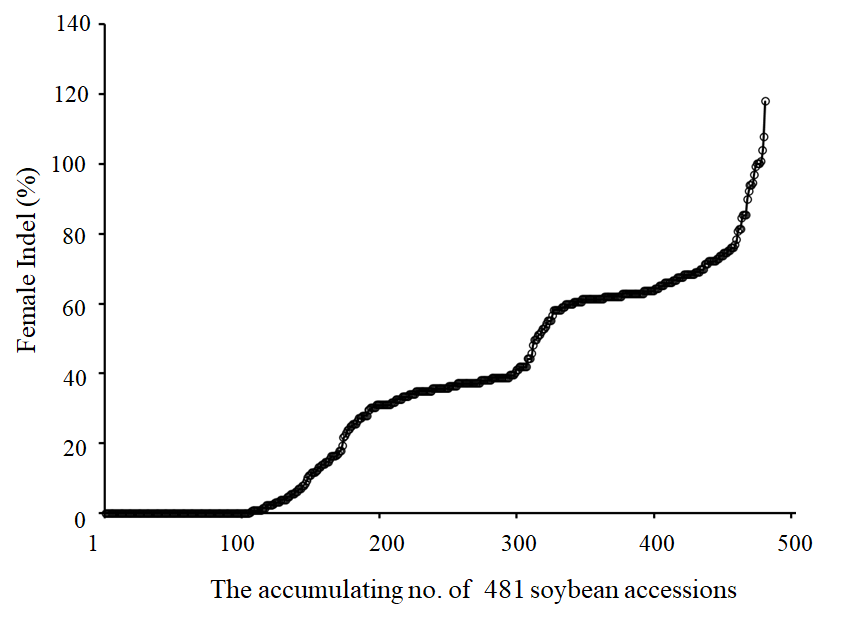

Supplement: Supplementary file 5 — Supplementary file5 (TIF 203 KB) [file 122_2023_4268_MOESM5_ESM.tif]

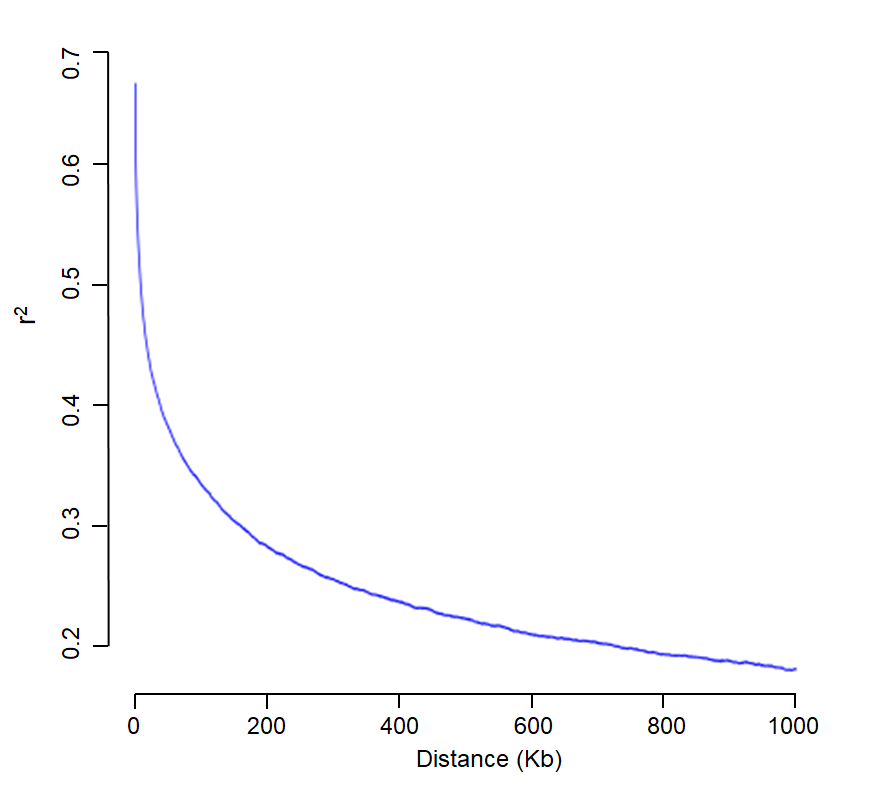

Supplement: Supplementary file 6 — Supplementary file6 (TIF 195 KB) [file 122_2023_4268_MOESM6_ESM.tif]

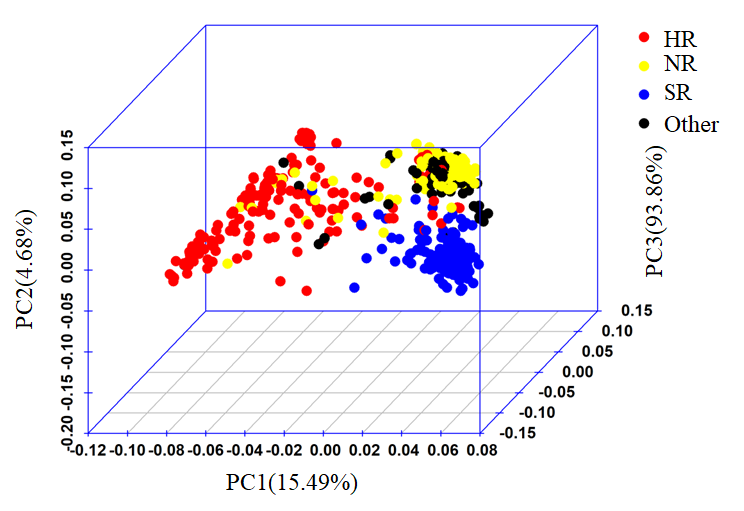

Supplement: Supplementary file 7 — Supplementary file7 (TIF 281 KB) [file 122_2023_4268_MOESM7_ESM.tif]

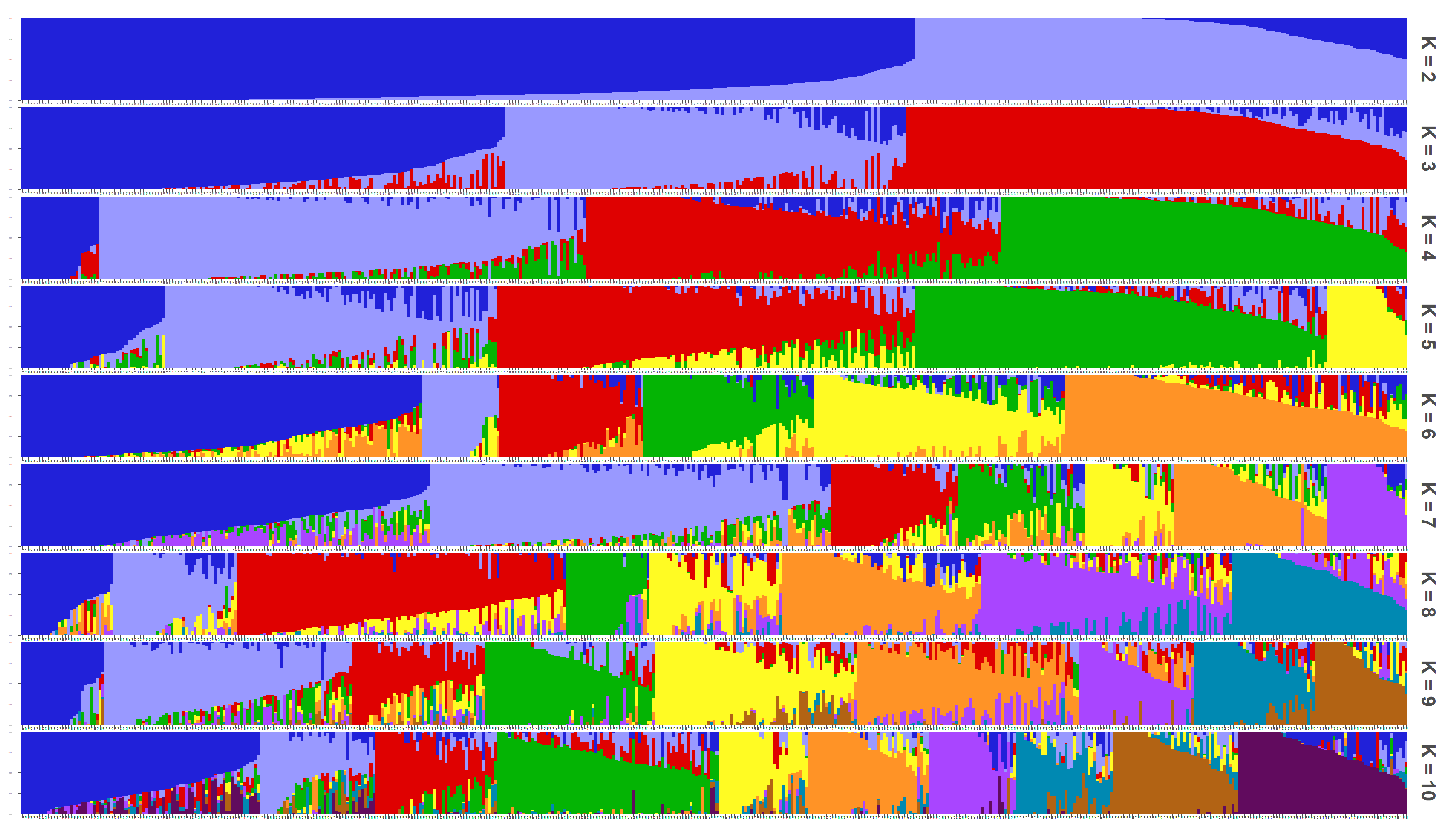

Supplement: Supplementary file 8 — Supplementary file8 (TIF 569 KB) [file 122_2023_4268_MOESM8_ESM.tif]

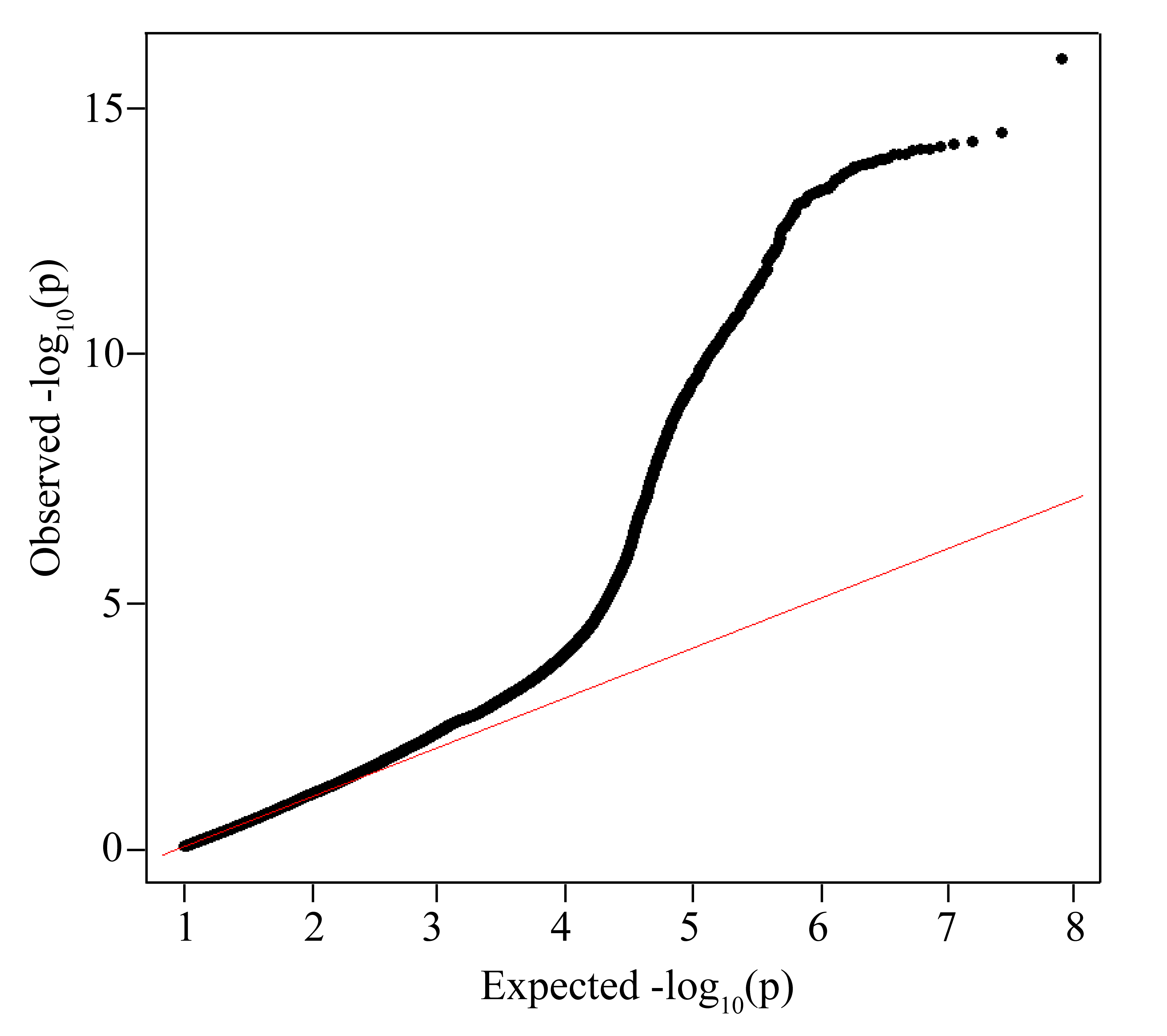

Supplement: Supplementary file 9 — Supplementary file9 (TIF 1549 KB) [file 122_2023_4268_MOESM9_ESM.tif]

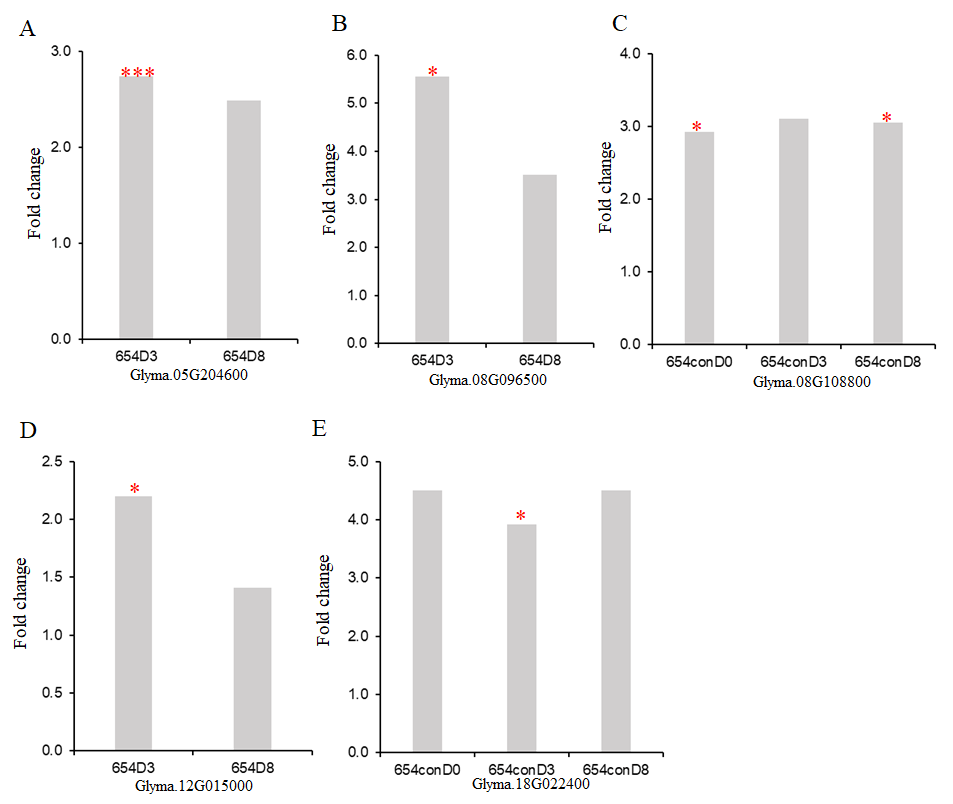

Supplement: Supplementary file 10 — Supplementary file10 (TIF 383 KB) [file 122_2023_4268_MOESM10_ESM.tif]

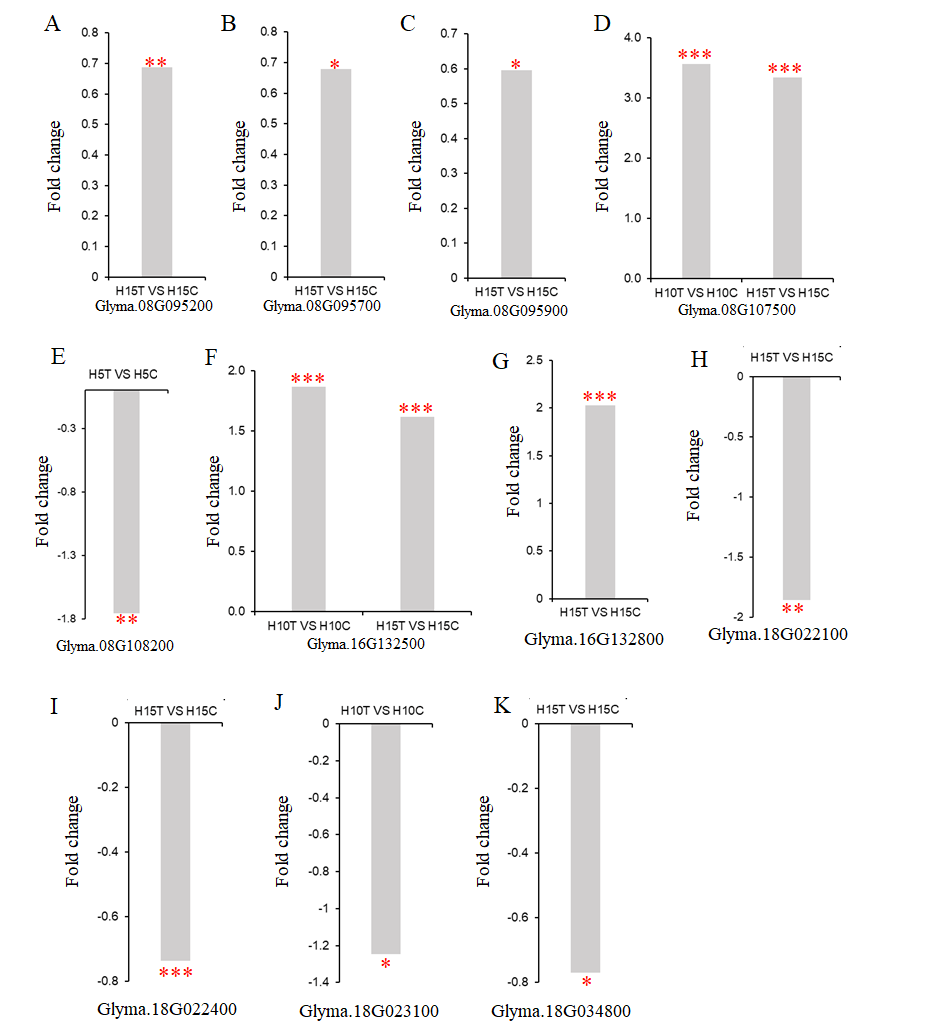

Supplement: Supplementary file 11 — Supplementary file11 (TIF 533 KB) [file 122_2023_4268_MOESM11_ESM.tif]

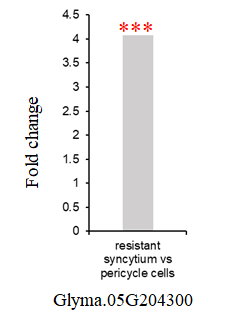

Supplement: Supplementary file 12 — Supplementary file12 (TIF 76 KB) [file 122_2023_4268_MOESM12_ESM.tif]

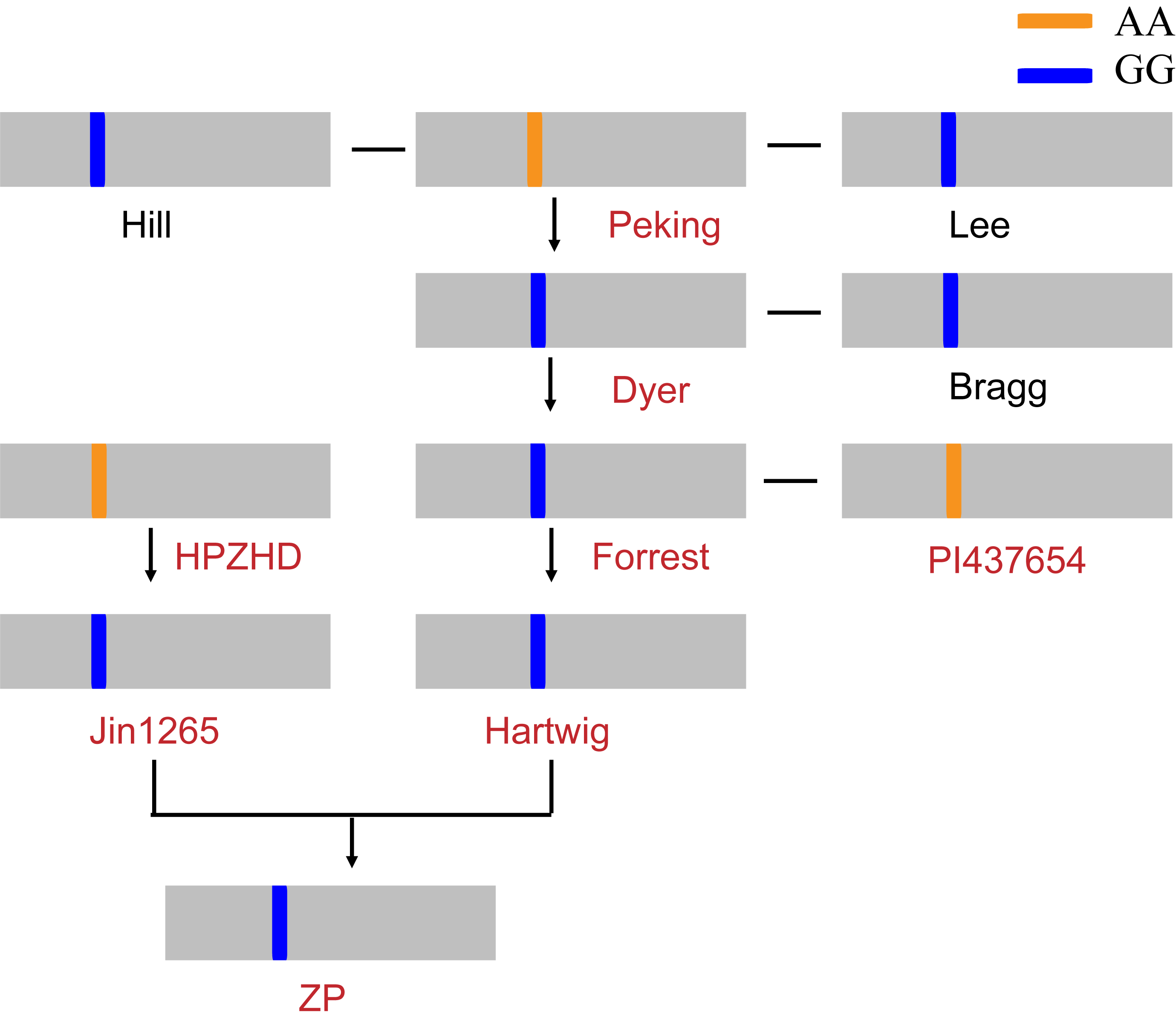

Supplement: Supplementary file 13 — Supplementary file13 (TIF 838 KB) [file 122_2023_4268_MOESM13_ESM.tif]

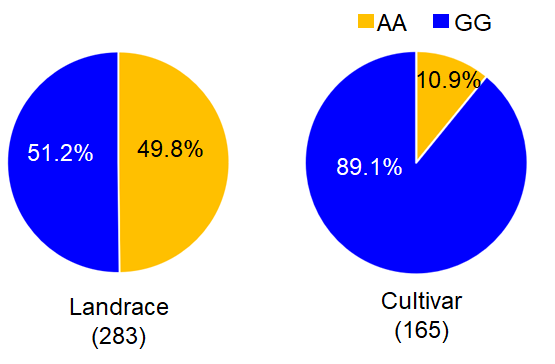

Supplement: Supplementary file 14 — Supplementary file14 (TIF 111 KB) [file 122_2023_4268_MOESM14_ESM.tif]

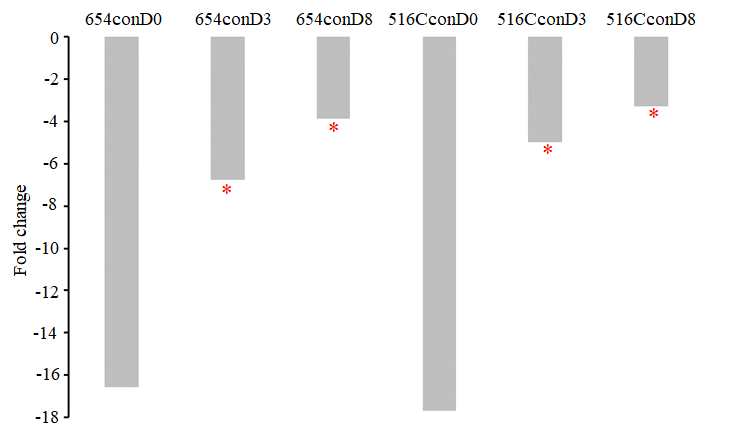

Supplement: Supplementary file 15 — Supplementary file15 (TIF 280 KB) [file 122_2023_4268_MOESM15_ESM.tif]
